# Anion-templated hexagonal nanotubes†
†Electronic supplementary information (ESI) available: Further details of synthesis and characterisation, crystal growth experiments, SCXRD and PXRD experiments, and anion binding studies. CCDC 1400481, 1400482, 1408282 and 1408283. For ESI and crystallographic data in CIF or other electronic format see DOI: 10.1039/c5sc02577j


**DOI:** 10.1039/c5sc02577j

**Published:** 2015-08-27

**Authors:** Nicholas G. White, Mark J. MacLachlan

**Affiliations:** a Department of Chemistry , University of British Columbia , 2036 Main Mall , Vancouver , BC V6T 1Z1 , Canada . Email: mmaclach@chem.ubc.ca

## Abstract

Hydrogen bonding between bromide anions and a tetrahydroxytriptycene ligand was used to assemble crystalline hexagonal tubes with nanometer diameters in good yield. Use of a hexahydroxytriptycene ligand again gave hexagonal nanotubes, but containing the spontaneously-oxidised quinone–tetrahydroxy ligand. The surprisingly robust nanotubes are stable to heat, vacuum and water, and represent an unprecedented use of O–H···anion coordination to assemble complex three-dimensional structures.

## Abstract 2

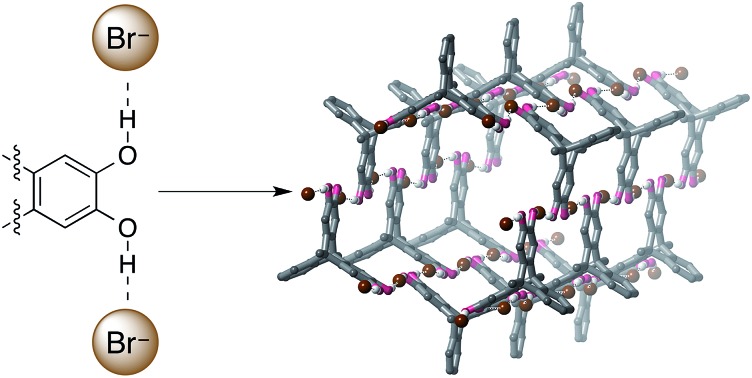
O–H···Br^–^ hydrogen bonding gives water-stable hexagonal nanotubes.

## Introduction

Inspired by Nature, directed self-assembly^1^ is an efficient method for the synthesis of complex three-dimensional systems, including framework materials,^2^ knots,^3^ and other topologically-intricate structures.^4^ These supermolecules have primarily been assembled using metal–ligand coordination bonds,^5^ although structures prepared through other non-covalent interactions, such as hydrophobic effects, aromatic stacking, and hydrogen bonding, are also known.^6^ In contrast, the use of anion coordination (whether hydrogen or halogen bonding to anions, or anion–π interactions) to form self-assembled systems is underexplored, presumably due to the difficulties associated with interacting with anions.^7^

Anion templation has been exploited within the field of transition metal chemistry, where the choice of counteranion to the metal cation can dramatically influence the structure of the product. A number of elegant examples of this approach have been reported,^8^ such as Lehn's helicates, where use of iron(ii) chloride gives a pentanuclear system containing a central chloride anion, while iron(ii) sulfate yields a hexanuclear product.8a,b Although such “counteranion” templation can give access to interesting complexes, the ability to prepare such products deliberately is limited, as any interaction between the anion and the system is much weaker than the metal–ligand interaction. Anion templation has also been used in the strategic synthesis of interlocked structures: an anion (typically a halide) is used to bring the two components together to form a precursor assembly, which is then turned into a permanently interlocked system by covalent modification.^9^

While the field is still very much in its infancy, recently a few studies have reported the use of anion templation to prepare self-assembled systems. Notably, the groups of Wu, and Kruger and Gunnlaugsson have prepared “tetranuclear” cages assembled through hydrogen bonding between four phosphate or sulfate anions and four bis-urea ligands.^10^ Other anion-templated cages, helicates, 1-D coordination polymers and 2-D layered structures have also been reported.^11^

**Fig. 1:**
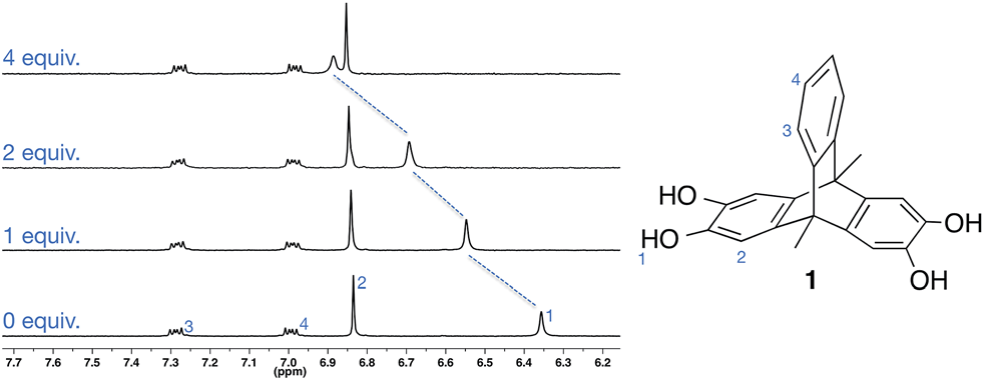
Portion of the ^1^H NMR spectrum of **1** on addition of TBA·Br (2.0 mM, CD_3_CN, 298 K).

Despite their prevalence in biological anion recognition processes,^12^ O–H···anion hydrogen bonds have received very little use in synthetic anion receptors.^13^ This is perhaps surprising given that hydroxyl groups can be potent hydrogen bond donors, and are often comparatively easy to synthesize. In an effort to investigate these under-utilized interactions, we have explored the anion-templated assembly of triptycene-containing tetrahydroxy ligand **1** (ref. 14) (Fig. 1). We demonstrate that O–H···anion hydrogen bonds are powerful structure–directing interactions and use them to form solid-state hexagonal^15^ nanotube architectures,^16^ which are remarkably stable.

## Results and discussion

### Solution anion binding of **1**

We initially investigated the solution anion binding behaviour of **1** using ^1^H NMR titration experiments in the polar organic solvent CD_3_CN. Aliquots of CD_3_CN solutions of anions as their TBA (tetrabutylammonium) salts were added to **1**, resulting in downfield shifts of the molecule's O–H resonance (Fig. 1).

Analysis of the titration data revealed that despite the presence of two catechol motifs, only one significant binding event was observed (unsubstituted catechol shows 1 : 1 binding with halide anions in CD_3_CN13b,c). Presumably this is because coordination of an anion to **1** deactivates the ligand, significantly reducing its affinity for a second anion. Fitting the titration data to a 1 : 1 binding model using either WinEQNMR2 (ref. 17) or Thordarson's *fittingprogram*^18^ gave 1 : 1 association constants, which are similar to those reported by Smith for unsubstituted catechol,13b,c and follow the trend Cl^–^ ≫ Br^–^ > I^–^ ∼ NO_3_^–^ (Table 1).^19^ Notably, the solution binding affinities of all anions are relatively modest.

**Table 1:** Association constants and approximate free energies of binding of tetrabutylammonium salts to **1** calculated using two different programs. Estimated standard errors of fitting^*a*^ given in parentheses

Anion	*K*_a_ ^*b*^ (M^–1^)	*K*_a_ ^*c*^ (M^–1^)	Δ*G*^*b*^ (kJ mol^–1^)	Δ*G*^*c*^ (kJ mol^–1^)
Cl^–^	558(18)	554(14)	–15.7	–15.7
Br^–^	85(5)	92(3)	–11.0	–11.2
I^–^	40(2)	43(3)	–9.1	–9.3
NO_3_^–^	37(1)	38(1)	–9.0	–9.0

^*a*^These are the errors in the fitting of the curve and are an approximate measure of the random error in the data. They do not account for systematic error (such as inaccuracies in the quantities of reagents measured out, or the temperature of the NMR spectrometer), and as such the true uncertainty is probably substantially larger.

^*b*^Determined using WinEQNMR2.^17^

^*c*^Determined using *fittingprogram*.^18^

### Solid-state structure of **1** and TBA·Br

Vapour diffusion of diethyl ether or pentane into mixtures of **1** and either one or two equivalents of TBA·Br in a wide range of solvents gave crystals. Single crystal X-ray diffraction (SCXRD) experiments showed that all of the crystals had extremely similar unit cells, despite the crystals being grown from a range of solvents (see ESI† for full details). Interestingly, we were not able to isolate any single crystalline products containing **1** when attempting to crystallize **1** with TBA·Cl, TBA·I, TBA·NO_3_ or TBA·HSO_4_, despite numerous attempts using several different solvent systems for each of these salts (see ESI† for further details).^20^,^21^

**Fig. 2:**
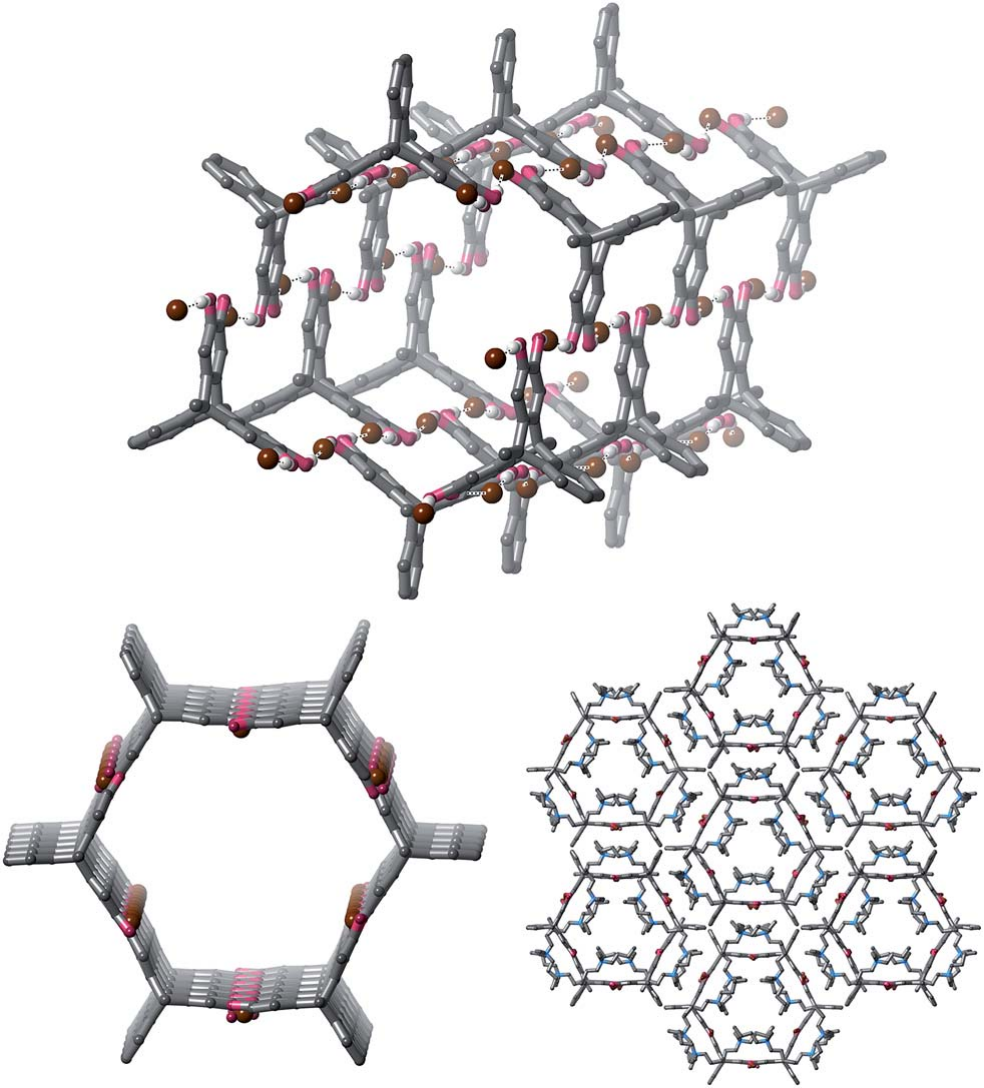
Views of the single crystal structure of [**1**·(TBA·Br)_2_]_*n*_. TBA cations and most hydrogen atoms are sometimes omitted for clarity.

Full structure determination of the crystals obtained from **1** and TBA·Br revealed that the product crystallizes as polymeric hexagonal nanotubes^22^ with the formula [**1**·(TBA·Br)_2_]_*n*_ (Fig. 2). The nanotubes are held together by short O–H···Br^–^ hydrogen bonds [O···Br: 3.038(8)–3.391(7) Å; H···Br: 2.19–2.54 Å, 72–83% of the sum of the van der Waal radii of H and Br^23^]. Each bromide anion receives two hydrogen bonds and the nanotubes have a face-to-face diameter of approximately 1.6 nm. The TBA counter-cations occupy the free spaces in the nanotube.

### Bulk synthesis of nanotubes

The nanotubes could be prepared in bulk by simply diffusing diethyl ether vapour into a 1 : 2 stoichiometric mixture of **1** and TBA·Br in acetonitrile. The product was isolated as single crystals in 67% yield after drying *in vacuo*.

**Fig. 3:**
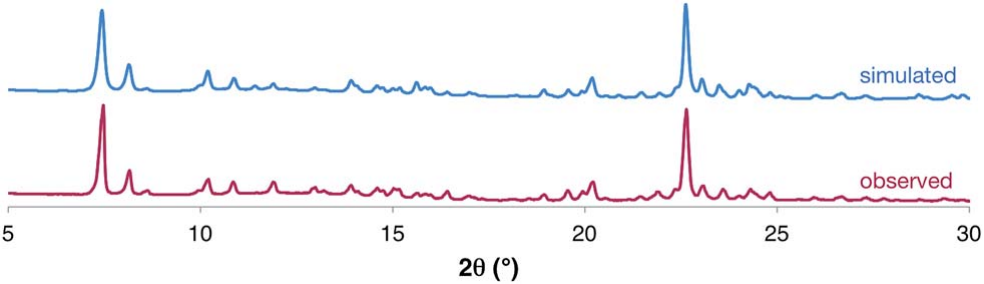
Comparison of the observed PXRD data for the dried bulk sample of [**1**·(TBA·Br)_2_]_*n*_ to that simulated from the SCXRD experiment. A more detailed analysis is provided in the ESI.†

Elemental analysis and ^1^H NMR spectroscopy confirmed the purity of the product, and powder X-ray diffraction (PXRD) of the dried bulk crystalline sample (Fig. 3) was used to demonstrate that the solid-state structure of the isolated product was consistent with the nanotubes identified by SCXRD studies. The product was further characterized by melting point analysis, IR spectroscopy and thermogravimetric analysis (see ESI†).

DOSY NMR spectroscopy in CD_3_CN was used to investigate the solution structure of the nanotubes (see ESI† for more details). No evidence for aggregation was observed, indicating that the nanotubes are broken apart upon dissolution.

### Stability of nanotubes

Despite the large number and large size of the cations, there is still significant void space in the crystalline structure. In the solid state, this space appears to be occupied by poorly-defined diffuse solvent molecules (see ESI† for more information). This solvent can be removed by drying *in vacuo* (as evidenced by ^1^H NMR spectroscopy, elemental analysis and thermogravimetric analysis), but disappointingly nitrogen adsorption measurements showed a negligible nitrogen-accessible surface area.^24^

**Fig. 4:**
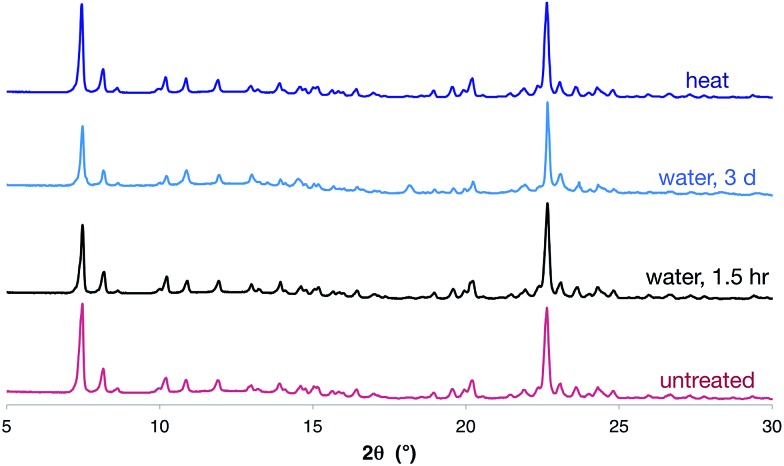
PXRD traces of [**1**·(TBA·Br)_2_]_*n*_ after soaking in water for 1.5 hours, three days, or after heating at ∼105 °C for 24 hours clearly showing that the nanotube structure is retained in all cases. The PXRD of an untreated sample of nanotubes is shown for comparison.

Importantly, the hexagonal nanotube structure remains intact upon drying in vacuum (as evidenced by SCXRD and PXRD), even though it is held together only by apparently-weak O–H···Br^–^ hydrogen bonds. Furthermore, the complex retains the crystalline nanotube structure, even after heating at ∼105 °C for 24 hours, or standing in water for three days (Fig. 4). This is remarkable given the modest solution binding strength measured between **1** and bromide anions, and may result in part from the hydrophobic character introduced by the triptycene motifs. We are unaware of any other materials assembled by anion coordination that have been reported to be stable in water over extended periods of time.

### Assembly of hexahydroxytriptycene with TBA·Br

**Fig. 5:**
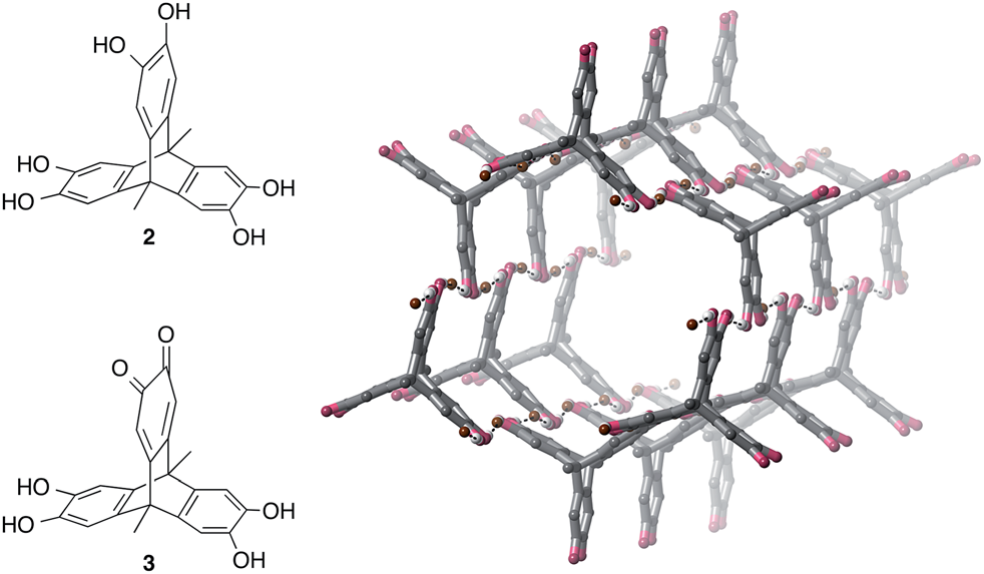
Structure of tris-catechol triptycene derivative **2**, the partially-oxidised quinone form **3**, and the crystal structure of nanotubes prepared from **3**, [**3**·(TBA·Br)_2_]_*n*_ (TBA cations and most hydrogen atoms omitted for clarity).

We next investigated whether a tris-catechol triptycene derivative **2** could be used to prepare anion-templated framework materials^25^ or honeycomb structures through O–H···Br^–^ hydrogen bonding (Fig. 5). Due to the low solubility of **2** in acetonitrile, we used methanol as solvent.^26^ Vapour diffusion of diethyl ether into a methanol solution of **2** and three equivalents of TBA·Br gave large darkly-coloured single crystals; however, instead of a 3D framework, these were surprisingly revealed to be hexagonal nanotubes with the formula [**3**·(TBA·Br)_2_]_*n*_, where **3** is a partially-oxidized form of **2** containing one quinone ring (Fig. 5, see ESI† for further details).

We have found that hexahydroxytriptycene **2** can be readily air-oxidized to give **3** in 85% yield by simply stirring in methanol in the presence of KOAc followed by aqueous work-up (see the ESI†). Vapour diffusion of diethyl ether into a methanol or acetonitrile solution of **3** and two equivalents of TBA·Br gave bulk samples of [**3**·(TBA·Br)_2_]_*n*_; ^1^H NMR spectroscopy, elemental analysis and PXRD showed that the bulk material was pure and had the nanotube structure observed by SCXRD. It is noteworthy that the nanotubes crystallize from the highly competitive hydrogen-bonding solvent methanol.

## Conclusions

In this work, we have demonstrated that relatively weak O–H···anion hydrogen bonding interactions (*K*_a_ ∼ 80 M^–1^ for Br^–^ in CD_3_CN) can be used to assemble complex three-dimensional nanotube structures. Even though these interactions are weak in solution, in the solid state, they act in concert to give stable materials that can be grown from methanol solution, or retain their structure even after soaking in water for several days. Importantly, these results demonstrate that interactions involving anions that are weak in solution can be used to prepare practically-useful solid-state materials. Work is continuing in our laboratories to expand this new strategy to prepare porous anion-templated materials.

## Experimental

### General remarks

Triptycene tetrol **1** (ref. 14) and triptycene hexol **2** (ref. 27) were both prepared in three steps from 1,2-dimethoxybenzene as previously described. The oxidation of **2** to **3** is described in the ESI.† All other reagents and solvents were bought from commercial suppliers and used as received.

### Synthesis of [**1**·(TBA·Br)_2_]_*n*_

Dimethyltetrahydroxytriptycene **1** (35 mg, 0.10 mmol) and TBA·Br (64 mg, 0.20 mmol) were dissolved in acetonitrile (5 mL) and subjected to diethyl ether vapour diffusion. Over 2–4 days, large brown crystals developed; these were isolated by filtration, washed with copious diethyl ether and thoroughly dried *in vacuo* to give [**1**·(TBA·Br)_2_]_*n*_ as brown single crystals. Yield: 66 mg (0.067 mmol, 67%). ^1^H NMR (CD_3_CN, 5.0 mM, 400 MHz): 7.26–7.30 (m, 2H), 6.97–7.01 (m, 2H), 6.85 (s, 4H), 6.82‡
‡Peak disappears on addition of D_2_O. (br. s, 4H), 3.05–3.10 (m, 16H), 2.21 (s, 6H), 1.56–1.64 (m, 16H), 1.30–1.39 (m, 16H), 0.97 (t, *J* = 7.3 Hz, 24H).

EA: C 65.7, H 9.4, N 2.7%; calc. for [**1**·(TBA·Br)_2_], C_54_H_90_N_2_O_4_Br_2_: C 65.4, H 9.2, N 2.8%. Mp: 140.0–141.5 °C. IR: ∼3180 cm^–1^ (broad, O–H stretch).

Product identity was elucidated using single crystal X-ray diffraction; powder X-ray diffraction on the bulk sample showed that the identity of the bulk product was consistent with the single crystal structure.

### Synthesis of [**3**·(TBA·Br)_2_]_*n*_

The tetrahydroxy quinone ligand **3** (19 mg, 0.050 mmol) and TBA·Br (32 mg, 0.10 mmol) were dissolved in methanol (5 mL) and subjected to diethyl ether vapour diffusion. Over approximately a week, very dark crystals developed; these were isolated by filtration, washed with copious diethyl ether and thoroughly dried *in vacuo* to give [**3**·(TBA·Br)_2_]_*n*_ as very dark brown single crystals. Yield: 17 mg (0.017 mmol, 34%).

^1^H NMR (CD_3_CN, 5.0 mM, 400 MHz): 7.34‡ (br. s, 4H), 6.90 (s, 4H), 6.07 (s, 2H), 3.05–3.11 (m, 16H), 2.04 (s, 6H), 1.54–1.63 (m, 16H), 1.29–1.39 (m, 16H), 0.96 (t, *J* = 7.3 Hz, 24H).

EA: C 63.2, H 8.7, N 2.6%; calc. for [**3**·(TBA·Br)_2_], C_54_H_88_N_2_O_6_Br_2_: C 63.5, H 8.7, N 2.7%. Mp: 193–195 °C. IR: ∼3160 (broad, O–H stretch), 1652 (C=O stretch) cm^–1^.

Product identity was elucidated using single crystal X-ray diffraction; powder X-ray diffraction on the bulk sample showed that the identity of the bulk product was consistent with the single crystal structure.

The synthesis could also be performed using acetonitrile instead of methanol as solvent to give product of indistinguishable purity in similar yield.

## Supplementary Material

Supplementary information

Crystal structure data
