# Supplementary material for: Anion-templated hexagonal nanotubes
Source: Chem Sci. 2015 Aug 27;6(11):6245–9. doi: 10.1039/c5sc02577j (PMC6054046; doi:10.1039/c5sc02577j)
Supplement: Supplementary file 1 [file SC-006-C5SC02577J-s001.zip › Supplementary Information.pdf]

Anion-templated hexagonal nanotubes

Nicholas G. White and Mark J. MacLachlan*

Department of Chemistry, University of British Columbia, 2036 Main Mall, Vancouver, British Columbia, Canada, V6T 1Z1

Email: mmaclach@chem.ubc.ca

Contents	1
Details of instrumentation	2
Synthesis and characterization	3
NMR spectra of new compounds	4
Details of crystallization experiments	5
Single crystal X-ray crystallography	7
Powder X-ray diffraction	12
Thermogravimetric analysis	16
Solution anion binding studies	17
DOSY NMR spectroscopy	19
References	23

Details of instrumentation

NMR spectra were recorded on Bruker AV-300 or Bruker AV-400 spectrometers and referenced to residual solvent signals.[S1] Melting points were recorded on a Stanford Research Systems Digimelt. TGA traces were recorded on a Perkin Elmer Pyris 6 instrument and IR spectra were recorded on a Thermo Scientific Nicolet 6700 FTIR spectrometer with an attenuated total reflectance (ATR) attachment. Elemental analyses (C,H,N) were performed at the UBC Microanalytical Services Laboratory. Single crystal X-ray diffraction (SCXRD) data were collected on a Bruker Apex DUO diffractometer at 90 K. Powder X-ray diffraction (PXRD) data were collected on a Bruker D8 Discover diffractometer at room temperature.

Synthesis and characterization

Synthesis of 3

Dimethylhexahydroxytriptycene **2** (38 mg, 0.10 mmol) was dissolved in methanol (5 mL). A solution of KOAc (20 mg, 0.20 mmol) in methanol (10 mL) was added to give a clear solution. This was stirred in a B24-necked 25 mL round-bottomed flask, open to the air, for 24 h. During this time, the solution turned a deep purple colour and the solvent evaporated to give a purple powder. This was partitioned between ethyl acetate (20 mL) and 1 M $HCl_{(aq)}$ (20 mL); the organic phase was washed with additional 1 M $HCl_{(aq)}$ (20 mL) and brine (20 mL), dried ($MgSO_4$), and taken to dryness to give **3** as a purple crystalline solid. Yield: 31 mg (0.081 mmol, 81%).

^{1}H NMR (CD_3CN, 400 MHz): 6.86 (s, 4H), 6.76* (s, 4H), 6.10 (s, 2H), 2.06 (s, 6H).
^{13}C NMR (CD_3CN, 100 MHz): 181.6, 157.9, 144.9, 135.1, 118.4, 110.4, 46.7, 14.1.
**Peak disappears on addition of D_2O*
HRESI-MS (pos.): 399.0847, calc. for $[C_{22}H_{16}O_6{\cdot}Na]^+$ = 399.0845. M. Pt.: > 260 ºC.
IR: ~ 3250 (broad, O–H stretch), 1635 (C=O stretch) cm^{-1}.

NMR spectra of new compounds

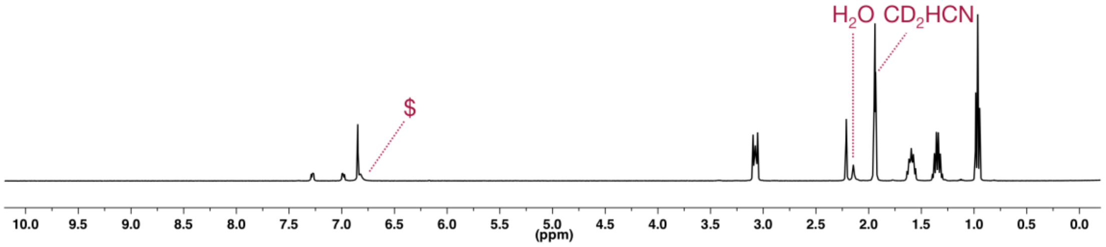

Figure S1. ^{1}H NMR spectrum of $[\mathbf{1}\cdot(\mathrm{TBA}\cdot\mathrm{Br})_2]_n$; peak labeled \$ disappears on addition of D_2O (5.0 mM in CD_3CN, 298 K, 400 MHz).

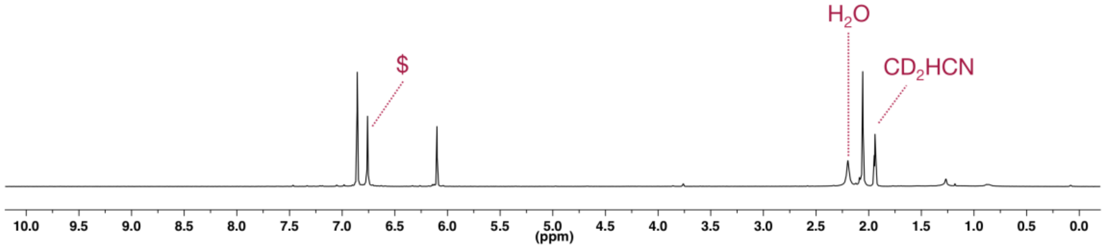

Figure S2. ^{1}H NMR spectrum of **3**; peak labeled \$ disappears on addition of D_2O (CD_3CN, 298 K, 400 MHz).

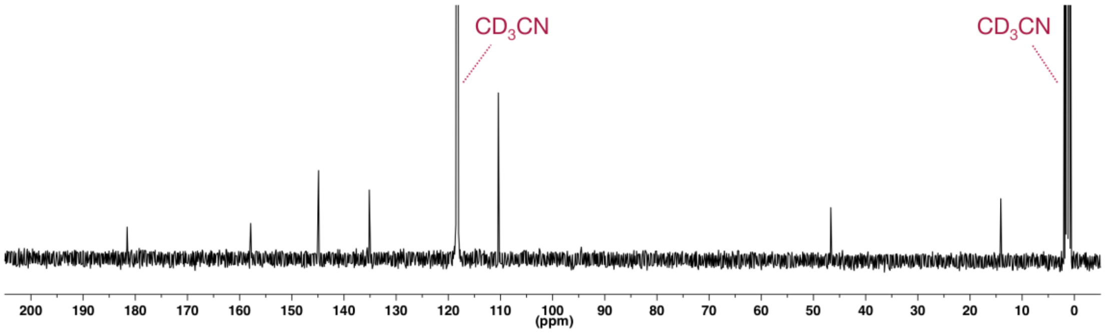

Figure S3. ^{13}C NMR spectrum of **3** (CD_3CN, 298 K, 100 MHz).

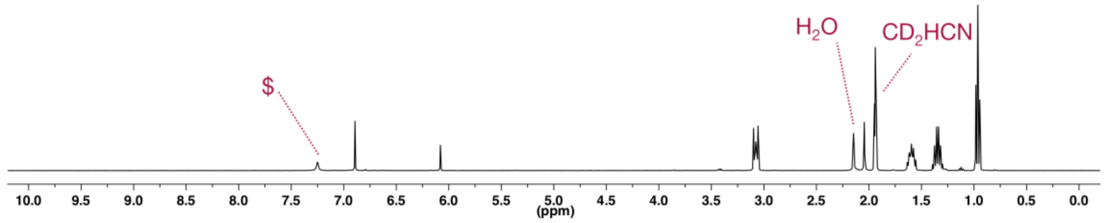

Figure S4. ^{1}H NMR spectrum of $[\mathbf{3}\cdot(\mathrm{TBA}\cdot\mathrm{Br})_2]_n$; peak labeled \$ disappears on addition of D_2O (5.0 mM in CD_3CN, 298 K, 400 MHz).

Details of crystallization experiments

Attempted crystallizations were conducted by dissolving ~ 0.010 mmol (~ 3.5 mg) of **1** and the stated number of equivalents of tetrabutylammonium·anion salt in the stated solvent or solvent mixture and subjecting these solutions to either diethyl ether or pentane vapor diffusion.

Successful crystallizations

***1** and TBA·Br:*

**Reagents**	**Solvent**	**Anti-solvent (vapor)**	**Outcome**
**1** and 1.0 equiv. TBA·Br	EtOAc	Et_2O	crystals[a]
**1** and 2.0 equiv. TBA·Br	EtOAc	Et_2O	crystals[a]
**1** and 2.0 equiv. TBA·Br	MeCN	Et_2O	crystals[a]
**1** and 2.0 equiv. TBA·Br	acetone	Et_2O	crystals[a]
**1** and 2.0 equiv. TBA·Br	DCM[b]	Et_2O	crystals[a]
**1** and 2.0 equiv. TBA·Br	DCM/THF	Et_2O	crystals[a]
**1** and 2.0 equiv. TBA·Br	DCM/EtOAc	Et_2O	crystals[a]
**1** and 2.0 equiv. TBA·Br	DCM/EtOAc	Et_2O	crystals[a,c]
**1** and 2.0 equiv. TBA·Br	DCM/THF	pentane	crystals[a]
**1** and 2.0 equiv. TBA·Br	DCM/EtOAc	pentane	crystals[a]

[a] Crystals had unit cell dimensions consistent with $[\mathbf{1}\cdot(TBA\cdot Br)_2]_n$
[b] **1** is insoluble in DCM, so the reagents were combined in acetone and taken to dryness under reduced pressure to give an oil, which was dissolved in DCM and subjected to Et_2O vapor diffusion.
[c] Crystals were dried under high vacuum for 8 hours and then diffraction data collected.

Unsuccessful crystallizations

Using MeOH as solvent:

***1** and TBA·Br*

**Reagents**	**Solvent**	**Anti-solvent (vapor)**	**Outcome**
**1** and 2.0 equiv. TBA·Br	MeOH	Et_2O	oil

<u>Other anions:</u>

***1** and TBA·Cl:*

Reagents	Solvent	Anti-solvent (vapor)	Outcome
**1** and 2.0 equiv. TBA·Cl	acetone	Et_2O	oil
**1** and 2.0 equiv. TBA·Cl	DCM/EtOAc	Et_2O	oil

***1** and TBA·I:*

Reagents	Solvent	Anti-solvent (vapor)	Outcome
**1** and 2.0 equiv. TBA·I	acetone	Et_2O	TBA·I crystals[a]
**1** and 2.0 equiv. TBA·I	DCM/EtOAc	Et_2O	TBA·I crystals[a]

[a] Crystals gave a unit cell matching a known structure of TBA·I.[S2]

***1** and TBA·NO_3:*

Reagents	Solvent	Anti-solvent (vapor)	Outcome
**1** and 2.0 equiv. TBA·NO_3	MeCN	Et_2O	oil
**1** and 2.0 equiv. TBA·NO_3	acetone	Et_2O	oil
**1** and 2.0 equiv. TBA·NO_3	DCM[a]	Et_2O	oil

[a] **1** is insoluble in DCM, so the reagents were combined in acetone and taken to dryness under reduced pressure to give an oil, which was dissolved in DCM and subjected to Et_2O vapor diffusion.

***1** and TBA·HSO_4:*

Reagents	Solvent	Anti-solvent (vapor)	Outcome
**1** and 2.0 equiv. TBA·HSO_4	MeCN	Et_2O	oil
**1** and 2.0 equiv. TBA·HSO_4	acetone	Et_2O	oil
**1** and 2.0 equiv. TBA·HSO_4	DCM[a]	Et_2O	oil

[a] **1** is insoluble in DCM, so the reagents were combined in acetone and taken to dryness under reduced pressure to give an oil, which was dissolved in DCM and subjected to Et_2O vapor diffusion.

Single crystal X-ray crystallography

Single crystal X-ray data were collected on a Bruker APEX DUO diffractometer using graphite monochromated Mo Kα radiation (λ = 0.71073 Å). All data were collected at 90 K to a resolution of 0.77 Å. Raw frame data (including data reduction, interframe scaling, unit cell refinement and absorption corrections) for all structures were processed using APEX2.[S4] Structures were solved using SUPERFLIP[S5] and refined using full-matrix least-squares on F^2 within the CRYSTALS suite.[S6] Unless otherwise stated, all non-hydrogen atoms were refined with anisotropic displacement parameters. Hydrogen atoms were generally visible in the Fourier difference map and were initially refined with restraints on bond lengths and angles, after which the positions were used as the basis for a riding model.[S7] Individual structures are discussed in more detail below. Full crystallographic data in CIF format are provided as Supporting Information [CCDC Numbers: 1400481, 1400482, 1408282, 1408283], as well as an annotated copy of the checkCIF output.

Structure of [1·(TBA·Br)$_2$]$_n$

Crystals of [**1**·(TBA·Br)$_2$]$_n$ while very large, tended to diffract poorly. Preliminary diffraction data were collected from numerous crystals grown from different solvents (as well as crystals that had been dried thoroughly *in vacuo*), but all with similar results. Three full datasets (from three different crystals) were collected, and all solved to give the same hexagonal nanotube structure; the highest quality dataset was fully refined and is presented here.

The structure crystallizes in the trigonal space group P3c1. The asymmetric unit cell contains three molecules of **1** and six TBA·Br cations, and the overall structure is a polymeric hexagonal nanotube (despite the high Z', no additional symmetry could be found). A small amount of diffuse electron density, believed to arise from disordered solvent molecules was also present. The data was handled in one of two ways, leading to two different refinements, which are discussed individually in the following paragraphs.

Fully squeezed refinement

Given the low quality of the data, and the difficulty in refining the ill-defined TBA cations, in this refinement (referred to in the CIF as "1_fullsqueeze," CSD No.: 1400481) the cations, as well as the small amount of diffuse electron density were included in the model using PLATON-SQUEEZE.[S8] It was necessary to add restraints to bond lengths and angles, and thermal and vibrational ellipsoid parameters, but this allowed a sensible refinement with all non-hydrogen atoms modeled anisotropically. Due to the low quality of the data phenolic hydrogen atoms could not be identified unambiguously, and so were inserted at idealized hydrogen bonding positions and these positions used as the basis for a riding model.

Partially squeezed refinement

As we were interested in the packing of the nanotube structures, we attempted to model the structure with the TBA cations included. This refinement is referred to in the CIF as "1_partialsqueeze," CSD No.: 1400482. A small amount of diffuse electron density, presumably arising from disordered solvent molecules was included in the model using PLATON-SQUEEZE.[S8] It was necessary to add restraints to bond lengths and angles, and thermal and vibrational ellipsoid parameters, but even with these restraints, it was not possible to achieve a chemically-sensible refinement, and so all atoms were refined isotropically. Due to the low quality of the data phenolic hydrogen atoms could not be identified unambiguously, and so were inserted at idealized hydrogen bonding positions and these positions used as the basis for a riding model.

Although the resulting structure is of low quality, bond lengths and angles refine to chemically-sensible parameters, and the overall structure of the assembly can be unambiguously determined.

Structure of [3·(TBA·Br)$_2$]$_n$ prepared from 2

This structure is referred to in the CIF as "oxidized_nanotubes_prepared_from_2" (CSD No.: 1408282).

Vapor diffusion of diethyl ether into a methanol solution of **2** and three equivalents of TBA·Br gave very large crystals of [**3**·(TBA·Br)$_2$]$_n$, where **2** has been oxidized to the mono-quinone form.

A small area of diffuse electron density is present, located on a special position (presumably arising from disordered solvent molecules). It was not possible to model this sensibly and so PLATON-SQUEEZE was used to include this electron density in the refinement. One of the TBA cations exhibits position disorder, this was modeled by having two positions for all of this cation's atoms. It was necessary to add restraints to the thermal and vibrational ellipsoid parameters of this disordered cation to achieve a sensible refinement; no restraints were added to the triptycene part of the molecule.

Assignment of quinone structure:

The SCXRD data unambiguously reveal that one of the three catechol moieties of **2** has been oxidized to the quinone form, as shown by a comparison of the bond lengths of this oxidized ring with one of the un-oxidized catechol rings (Figure S5). The bond lengths in the SCXRD structure of [**3**·(TBA·Br)$_2$]$_n$ prepared directly from **3** are very similar.

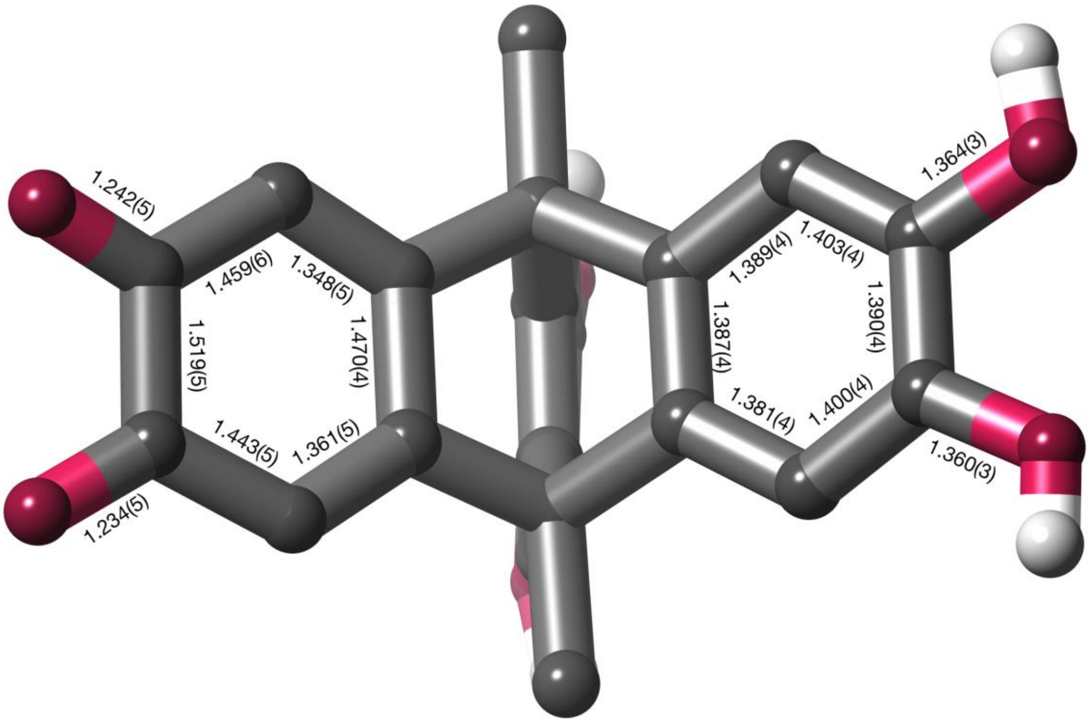

Figure S5. Comparison of bond lengths in oxidized quinone moiety and catechol moiety of **3** in the structure of [**3**·(TBA·Br)$_2$]$_n$ (prepared from hexahydroxy ligand **2**). All distances are reported in Å. Bond lengths in the other catechol moiety (obscured in the picture) are very similar to the catechol shown: C–C, 1.385(5)–1.404(4) Å; C–O, 1.367(4) and 1.377(4) Å .

Structure of [3·(TBA·Br)$_2$]$_n$ prepared directly from 3

This structure is referred to in the CIF as "oxidized_nanotubes_prepared_from_3" (CSD No.: 1408283).

Crystals of [**3**·(TBA·Br)$_2$]$_n$ diffracted well, although despite long exposure times (240 s/°), high angle data were quite weak. A small area of diffuse electron density is present, located on a special position (presumably arising from disordered solvent molecules). It was not possible to model this sensibly and so PLATON-SQUEEZE was used to include this electron density in the refinement. It was necessary to apply restraints to the bond lengths and angles, and vibrational and thermal ellipsoid parameters of the TBA cations to achieve a chemically-sensible refinement. No restraints were necessary on the triptycene part of the molecule. Hydroxyl protons could not be identified unambiguously in the difference map, and so are inserted at idealized hydrogen bonding positions.

Powder X-ray diffraction

Bulk samples of [**1**·(TBA·Br)$_2$]$_n$ and [**3**·(TBA·Br)$_2$]$_n$. were prepared In each case, these bulk samples were divided into two approximately equal halves, one half was used for PXRD experiments and the other used for other analytical techniques (EA, IR, thermogravimetric analysis etc.).

The observed spectra were recorded on vacuum-dried samples at room temperature, and are compared with the powder pattern calculated from the single crystal structures In the case of [**1**·(TBA·Br)$_2$]$_n$, the PXRD data were compared with the structure where PLATON-SQUEEZE[S8] was used only to remove presumed disordered solvents (and not poorly-behaved TBA cations). See the *single crystal X-ray crystallography* of the Supporting Information for further details regarding the refinement of these single crystal structures.

As the PXRD data were recorded at room temperature and the SCXRD data at 90 K, Rietveld refinement in TOPAS[S9] was used to refine the unit cell axis lengths and angles. In each case, the SCXRD unit cell dimensions increased by a small amount upon refinement (as would be expected, due to the ~ 200 K increase in temperature, see Tables S1 and S2). Rietveld refinement was also used to include corrections for small amounts of amorphous scattering (see Figures S6 to S9).

The following Figures show the observed PXRD data as well as the calculated simulation after allowing the unit cell dimensions and amorphous scattering component to refine; the "uncorrected" simulation is also included. For both [**1**·(TBA·Br)$_2$]$_n$ and [**3**·(TBA·Br)$_2$]$_n$, good agreement is observed between the PXRD traces of the thoroughly dried bulk samples and that simulated from the nanotube SCXRD structures, confirming that the bulk samples retain the extended 3D structure observed in the single crystals.

PXRD of [1·(TBA·Br)$_2$]$_n$

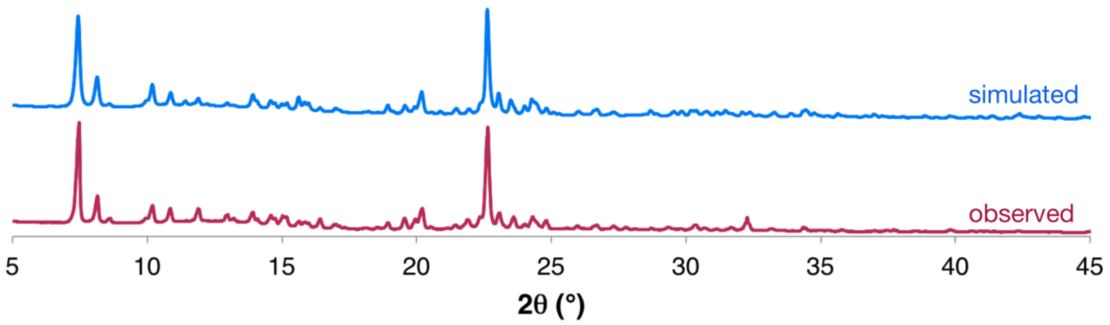

Figure S6. Comparison of observed and corrected simulated PXRD traces for [**1**·(TBA·Br)$_2$]$_n$.

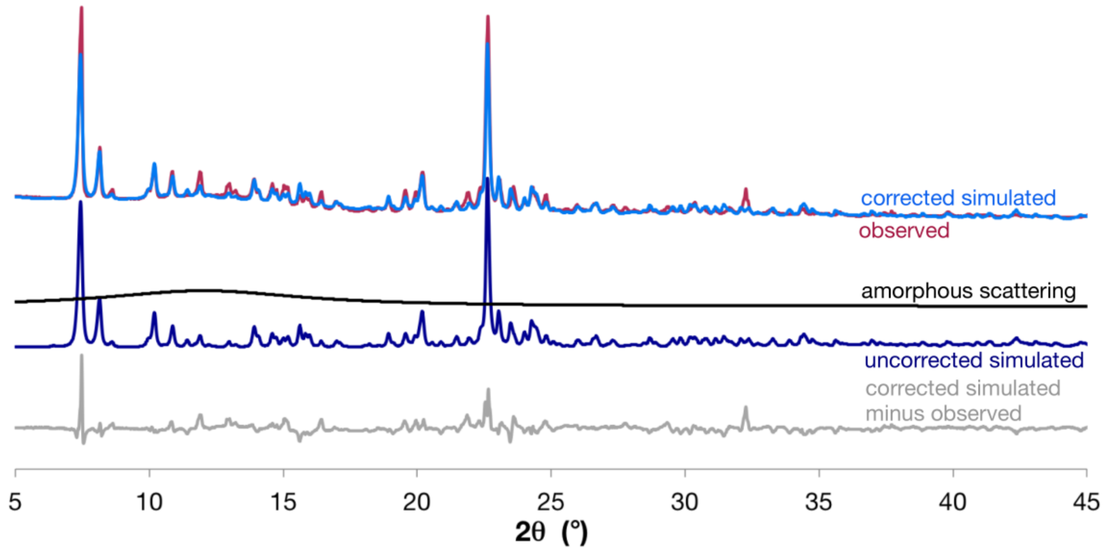

Figure S7. Comparison of observed and corrected simulated PXRD traces for [**1**·(TBA·Br)$_2$]$_n$, showing the uncorrected simulated trace, the calculated amorphous scattering correction and the difference between the corrected simulated and observed trace.

Table S1. Comparison of observed unit cell parameters in the SCXRD structure of [**1**·(TBA·Br)$_2$]$_n$ (at 90 K), and those calculated for the room temperature structure using Rietveld refinement of the PXRD data.

**Parameter**	**SCXRD (90 K)**	**PXRD (RT)**
	**Space group: P3c1**	
a = b	40.346(14)	40.7
c	17.984(6)	18.2
$\alpha = \beta$	90	*not refined*
γ	120	*not refined*

PXRD of [3·(TBA·Br)$_2$]$_n$

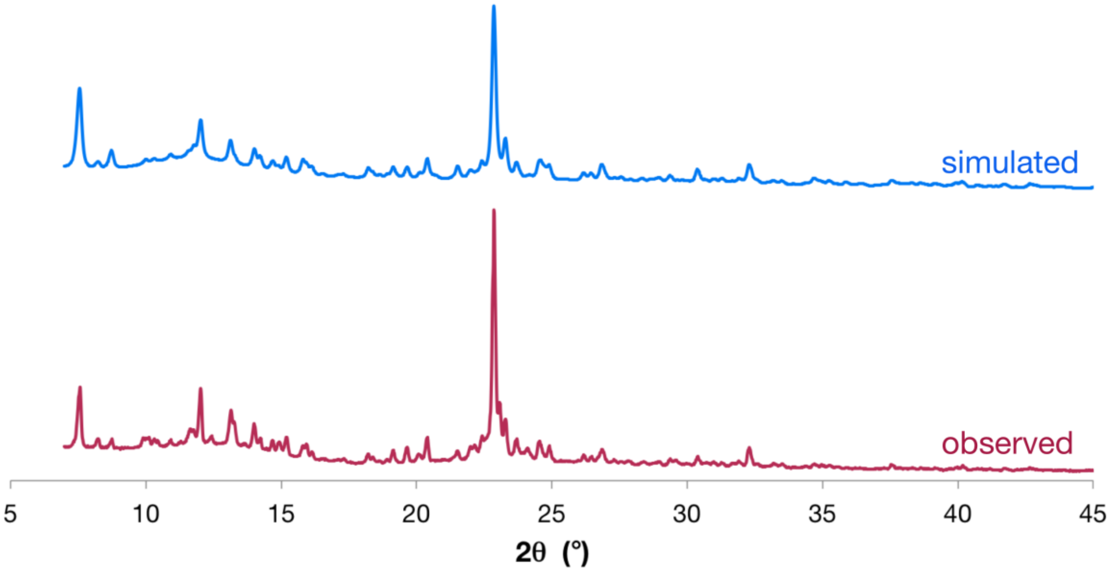

Figure S8. Comparison of observed and corrected simulated PXRD traces for [**3**·(TBA·Br)$_2$]$_n$.

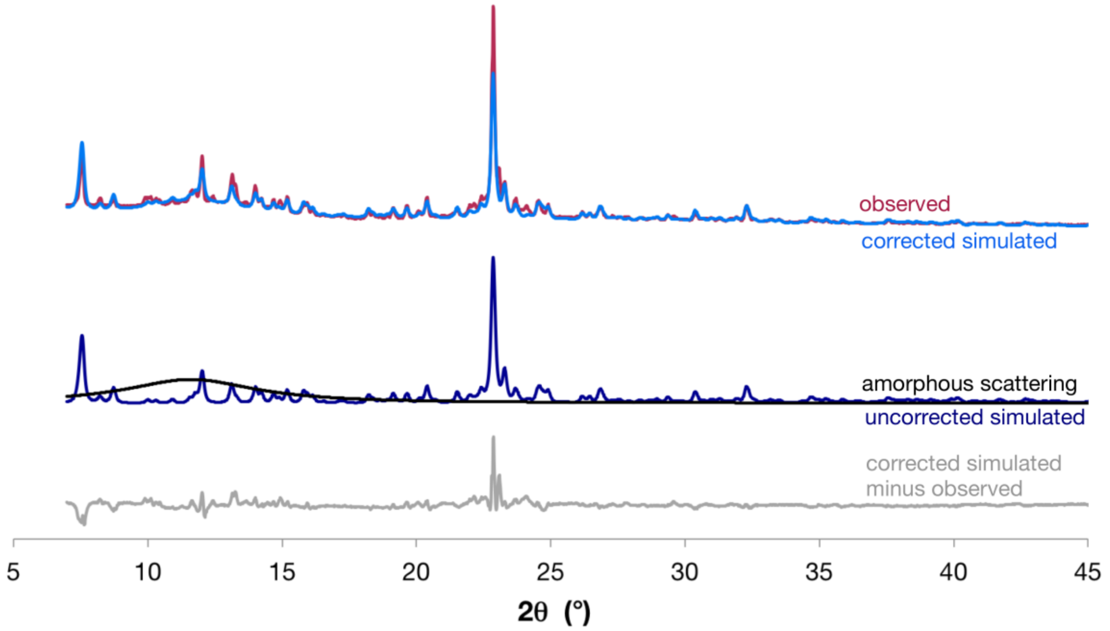

Figure S9. Comparison of observed and corrected simulated PXRD traces for [**3**·(TBA·Br)$_2$]$_n$, showing the uncorrected simulated trace, the calculated amorphous scattering correction and the difference between the corrected simulated and observed trace.

Table S2. Comparison of observed unit cell parameters in the SCXRD structure of [**3**·(TBA·Br)$_2$]$_n$ (at 90 K), and those calculated for the room temperature structure using Rietveld refinement of the PXRD data.

**Parameter**	**SCXRD (90 K)**	**PXRD (RT)**
	**Space group: R3c**	
a = b	39.896(4)	40.4
c	18.0796(17)	18.2
α = β	90	*not refined*
γ	120	*not refined*

Stability testing of [1·(TBA·Br)$_2$]$_n$

Heat testing

A small sample of [**1**·(TBA·Br)$_2$]$_n$ (~ 15 mg) was placed in a small glass vial. It was placed in an oven, left open to the air, for 24 hours. The temperature of the oven was set at 110 ºC, although a thermometer placed in the oven read ~ 105 ºC. After 24 hours, the sample was removed, cooled to room temperature, and analyzed by PXRD.

Water testing

Small samples of [**1**·(TBA·Br)$_2$]$_n$ (12–15 mg) were placed in glass vials. Water (~ 5 mL) was added to completely cover the crystals, the vials were capped and left to stand for either 1.5 hours or 3 days. The crystals were then isolated by filtration, washed with water (2 × 3 mL), air dried, dried *in vacuo*, and then analyzed by PXRD.

Thermogravimetric analysis

As shown in Figure S10, [**1**·(TBA·Br)$_2$]$_n$ shows reasonably high thermal stability, with thermal decomposition commencing at approximately 230 °C. The very slight mass loss (~ 1%) observed between 150 and 230 °C may be due to release of trace amounts of water and/or gases encapsulated within the crystalline lattice (M. Pt. of this compound = 140.0–141.5 °C).

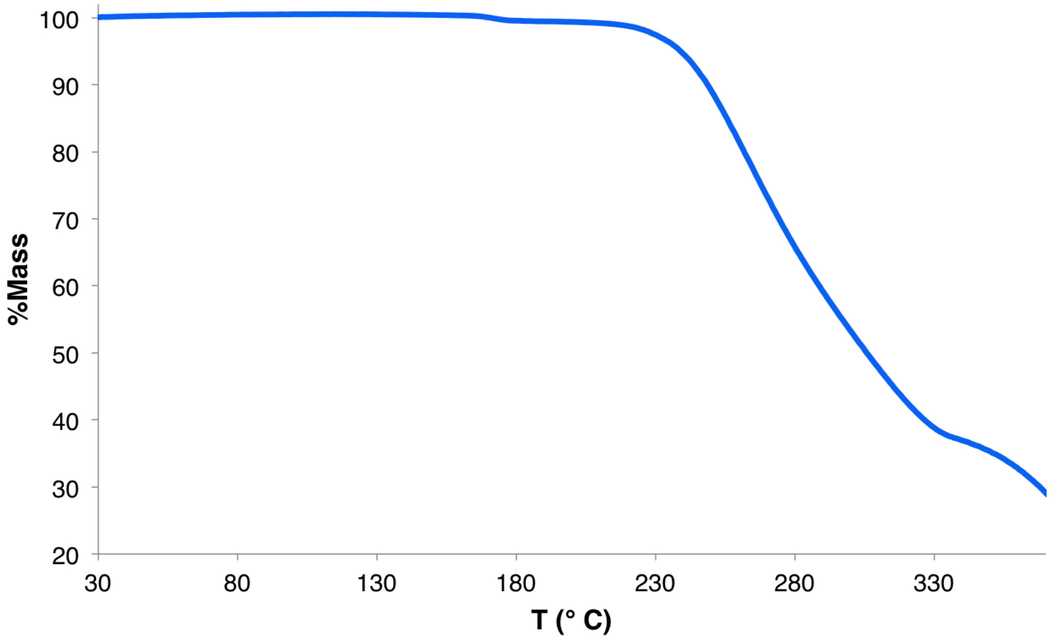

Figure S10. Thermogravimetric analysis plot of [**1**·(TBA·Br)$_2$]$_n$.

Solution anion binding studies

General protocol

All anion binding titration experiments were conducted in CD_3CN at 298 K. Initial sample volumes were 0.50 mL and concentrations were 2.0 mmol L^{-1} of host. Solutions (100 mmol L^{-1}) of anions as tetrabutylammonium salts were added in aliquots, the samples thoroughly shaken and spectra recorded. Spectra were recorded at 0, 0.2, 0.4, 0.6, 0.8, 1.0, 1.2, 1.4, 1.6, 1.8, 2.0, 2.2, 2.4, 2.6, 2.8, 3.0, 3.5, 4.0, 5.0, 6.0, 8.0 and 10 equivalents (Figure S11). Stability constants were obtained by analysis of the resulting data using WinEQNMR2[S10] and *fittingprogram*,[S11] following the O–H resonance in each case (no other resonances showed significant movement over the course of the titration experiments). Association constants show good consistency between the two fitting programs.

Binding stoichiometries

Intuitively, it may be expected that **1** would bind anions with a 1:2 receptor:anion stoichiometry (as unsubstituted catechol binds to halides with a 1:1 stoichiometry,[S12] and **1** contains two catechol motifs). Despite several attempts using both WinEQNMR2[S10] and *fittingprogram*,[S11] it was not possible to satisfactorily fit the binding data to a 1:2 ligand:anion model (unstable fitting refinements, estimated errors several times larger than the calculated association constants). In contrast, good 1:1 fits could be readily and reproducibly obtained. By visual inspection, the data are consistent with 1:1 binding, and so we suggest that is the dominant interaction in solution. Given that binding of the first anion is relatively modest, it is perhaps unsurprising that this deactivates the ligand such that binding of a second anion (in solution) is so weak as to be negligible.

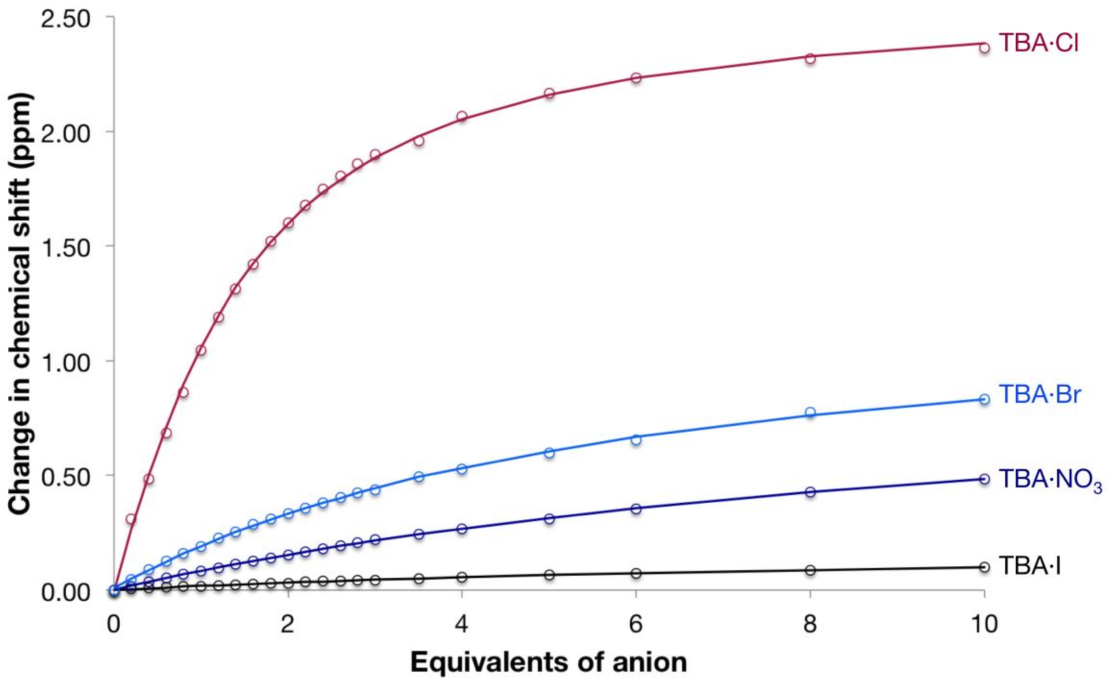

Figure S11. Movement of O–H resonance of **1** upon addition of tetrabutylammonium salts; circles represent data points, lines represent binding isotherms calculated using WinEQNMR2[S10] (CD_3CN, 298 K).

DOSY NMR spectroscopy of $[1\cdot(TBA\cdot Br)_2]_n$

DOSY NMR spectroscopy was performed on a 1:2 mixture of **1** and TBA·Br in CD_3CN (5.0 mM of **1** and 10 mM of TBA·Br). One molar equivalent of tetramethoxydimethyltriptycene was included as a reference compound (as its ability to hydrogen bond to either **1** or Br^- is expected to be negligible in polar CD_3CN). Diffusion coefficients were calculated using well-isolated resonances (see Figures S12 and S13), and are displayed in Table S3. Noticeably all diffusion coefficients show excellent concordance with other peaks from the same molecules (within 0.6%).

It is clearly apparent that **1** has a slightly smaller diffusion coefficient than the tetramethoxy compound, which is again smaller than the TBA cation. As the diffusion coefficient is inversely proportional to the (hydrodynamic) radius of the species, this demonstrates that **1** occupies a slightly smaller volume than the tetramethoxy reference, and is also smaller than the TBA cation. This is consistent with **1** existing as a discrete monomeric species in solution (possibly with an associated bromide anion), but rules out the possibility of significantly aggregated species.

Table S3. Peak positions and assignments, and diffusion coefficients, of selected resonances in the DOSY NMR experiment. Peak assignments refer to compound numbering in Figure S12. Curves fitted to calculate diffusion coefficients are displayed in Figure S13.

**Peak position (ppm)**	**Peak assignment**	**Diffusion coefficient ($m^2\ s^{-1}$)**
7.37	Tetramethoxy reference, resonance d	1.453×10^{-9}
7.30	Compound **1**, resonance 4	1.348×10^{-9}
7.04	Tetramethoxy reference, resonance b	1.445×10^{-9}
6.90	Compound **1**, resonance 2	1.344×10^{-9}
3.80	Tetramethoxy reference, resonance a	1.448×10^{-9}
3.09	TBA cation, N–CH_2	1.664×10^{-9}

Diffusion coefficients for each species in solution:

*Compound **1**:* 1.344, 1.348 × 10^{-9} m^2 s^{-1} (*Mean: 1.35 × 10^{-9} m^2 s^{-1}*)

Tetramethoxy reference: 1.445, 1.448, 1.453 × 10^{-9} m^2 s^{-1} (*Mean: 1.45 × 10^{-9} m^2 s^{-1}*)

TBA cation: 1.664 × 10^{-9} m^2 s^{-1}

Approximate relative hydrodynamic radii (with tetramethoxy reference defined as 1.00):

*Compound **1**:* 0.93

Tetramethoxy reference: 1.00

TBA cation: 1.15

These values suggest that **1** has a volume approximately 80% of that of the tetramethoxy reference in solution, while the TBA cation has a volume approximately 1.5 times larger than the reference. Given the rigid, non-spherical shapes of the triptycene compounds, these values are extremely approximate.

For a discussion of the benefits of determining the value of hydrodynamic radii/volumes by comparison with a reference compound (rather than the absolute value of these parameters), see Reference S13.

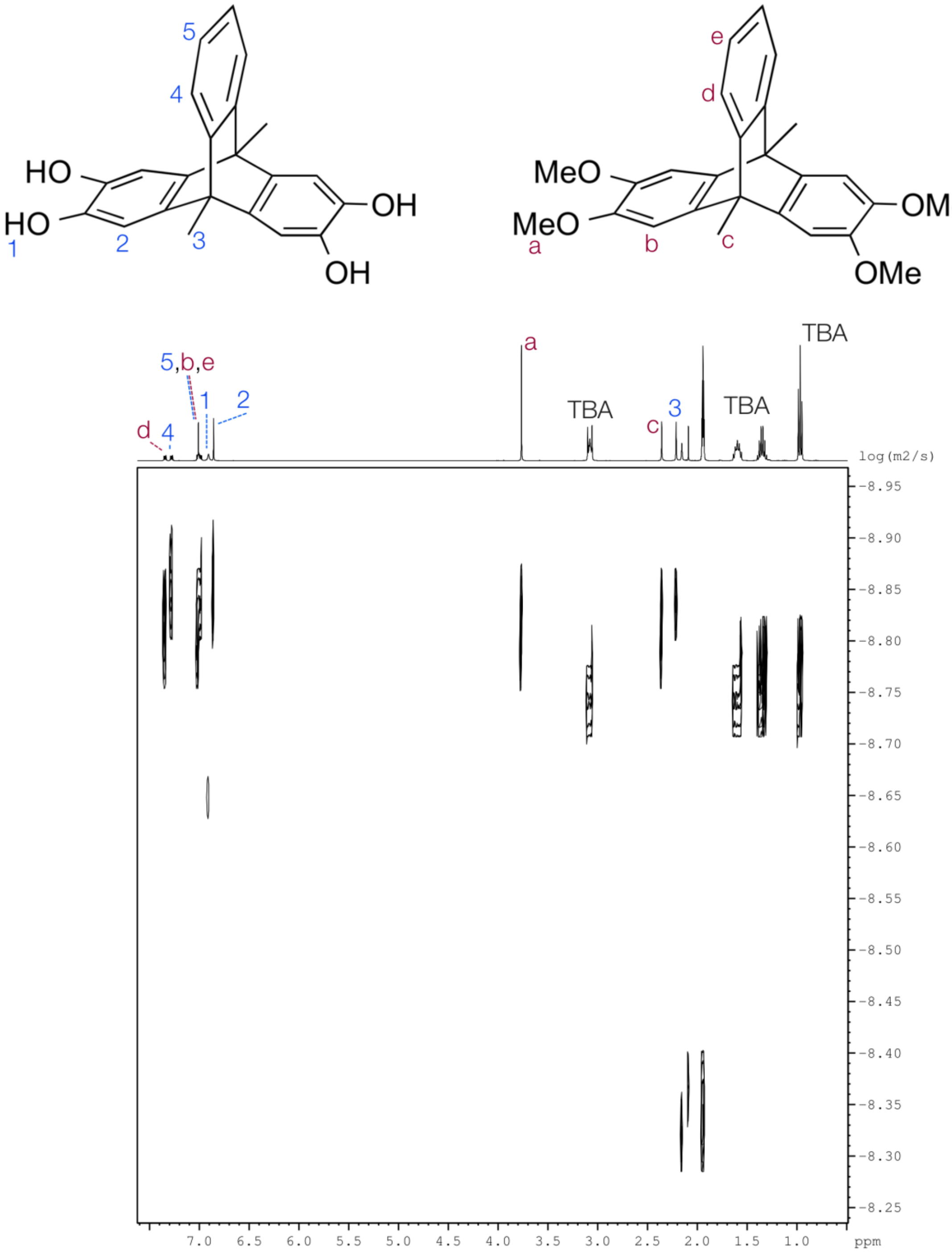

Figure S12. 2D DOSY NMR spectrum of 1:1:2 mixture of tetramethoxydimethyltriptycene, **1** and TBA·Br in CD_3CN (400 MHz, 298 K, 5.0 mM in **1**).

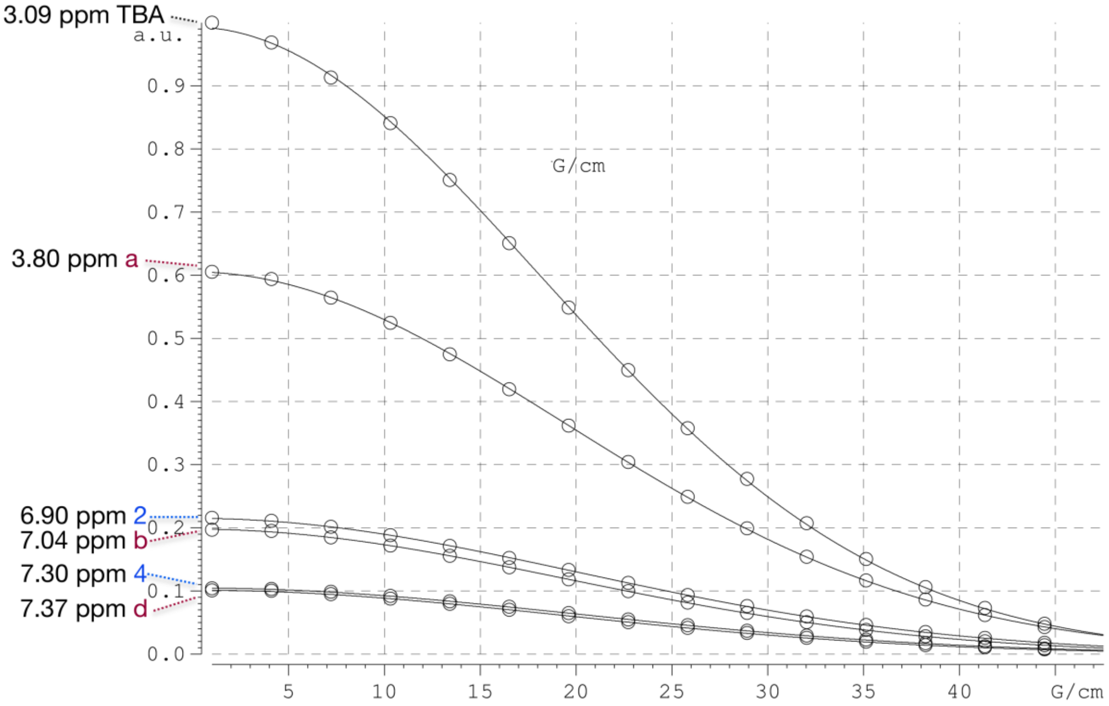

Figure S13. Graph showing observed (circles) and fit (lines) of peak intensity against gradient.

References

[S1] H. E. Gottlieb, V. Kotlyar, A. Nudelman, *J. Org. Chem.* **1997**, *62*, 7512–7515.

[S2] W. Prukala, B. Marciniec, M. Kubicki, *Acta Crystallogr.* **2007**, *E63*, o1464–o1466.

[S3] A. R. Campanelli, L. Scaramuzza, *Acta Crystallogr.* **1986**, *C42*, 1380–1383.

[S4] *APEX2*, **2007**, Bruker AXS Inc., Madison, Wisconsin, USA.

[S5] L. Palatinus, G. Chapuis, *J. Appl. Crystallogr.* **2007**, *40*, 786–790.

[S6] P. W. Betteridge, J. R. Carruthers, R. I. Cooper, K. Prout, D. J. Watkin, *J. Appl. Crystallogr.* **2003**, *36*, 1487.

[S7] R. I. Cooper, A. L. Thompson, D. J. Watkin, *J. Appl. Crystallogr.* **2010**, *43*, 1100–1107.

[S8] P. van der Sluis, A. L. Spek, *Acta Crystallogr.* **1990**, *A46*, 194–201; A. Spek, *J. Appl. Crystallogr.* **2003**, *36*, 7–13.

[S9] *TOPAS-Academic*, **2012**, Coelho Software, Brisbane, Australia.

[S10] M. J. Hynes, *J. Chem. Soc. Dalton Trans.* **1993**, 311–312.

[S11] P. Thordarson, *Chem. Soc. Rev.* **2011**, *40*, 1305–1323.

[S12] D. K. Smith, *Org. Biomol. Chem.* **2003**, *1*, 3874–3877; K. J. Winstanley, A. M. Sayer, D. K. Smith, *Org. Biomol. Chem.* **2006**, *4*, 1760–1767.

[S13] R. Neufeld, D. Stalke, *Chem. Sci.* **2015**, *6*, 3354–3364.
