# Supplementary material for: Anion-templated hexagonal nanotubes
Source: Chem Sci. 2015 Aug 27;6(11):6245–9. doi: 10.1039/c5sc02577j (PMC6054046; doi:10.1039/c5sc02577j)

Anion-templated hexagonal nanotubes

Nicholas G. White and Mark J. MacLachlan*

Department of Chemistry, University of British Columbia, 2036 Main Mall, Vancouver, British Columbia, Canada, V6T 1Z1

Email: mmaclach@chem.ubc.ca

Annotated CIFcheck

For a description of single crystal X-ray crystallography and detailed description of each structure, see the main Supporting Information document

checkCIF/PLATON report

You have not supplied any structure factors. As a result the full set of tests cannot be run.

THIS REPORT IS FOR GUIDANCE ONLY. IF USED AS PART OF A REVIEW PROCEDURE FOR PUBLICATION, IT SHOULD NOT REPLACE THE EXPERTISE OF AN EXPERIENCED CRYSTALLOGRAPHIC REFEREE.

Nanotubes prepared from **1**, where PLATON-SQUEEZE was used to include electron density from TBA cations (see main Supporting Information document for more information)

No syntax errors found. CIF dictionary Interpreting this report

Datablock: 1_fullsqueeze

```
Bond precision:   C-C = 0.0104 A          Wavelength=0.71073

Cell:              a=40.346(14)      b=40.346(14)      c=17.984(6)
                   alpha=90          beta=90           gamma=120
Temperature:       90 K

                  Calculated                  Reported
Volume            25352(23)                   25352(15)
Space group       P 3 c 1                     P 3 c 1
Hall group        P 3 -2"c                    P 3 -2"c
Moiety formula    C22 H18 O4, 2(Br)           3(C22 H18 O4), 6(Br)
Sum formula       C22 H18 Br2 O4              C66 H54 Br6 O12
Mr                506.16                      1518.57
Dx,g cm-3         0.597                       0.597
Z                 18                          6
Mu (mm-1)         1.448                       1.448
F000              4536.0                      4536.0
F000'             4527.89
h,k,lmax          50,50,22                    43,50,22
Nref              34441[ 17233]               34142
Tmin,Tmax         0.600,0.667                 0.623,1.000
Tmin'             0.381

Correction method= # Reported T Limits: Tmin=0.623 Tmax=1.000
AbsCorr = MULTI-SCAN

Data completeness= 1.98/0.99       Theta(max)= 26.331

R(reflections)= 0.1187( 25363)     wR2(reflections)= 0.2640( 26467)

S = 0.944                  Npar= 758
```

The following ALERTS were generated. Each ALERT has the format
test-name_ALERT_alert-type_alert-level.
Click on the hyperlinks for more details of the test.

Alert level B

```
PLAT213_ALERT_2_B Atom O108            has ADP max/min Ratio .....       4.4 prolat
```

Alert level C

```
DIFMN02_ALERT_2_C  The minimum difference density is < -0.1*ZMAX*0.75
            _refine_diff_density_min given =     -3.170
            Test value =     -2.625
DIFMN03_ALERT_1_C  The minimum difference density is < -0.1*ZMAX*0.75
            The relevant atom site should be identified.
DIFMX01_ALERT_2_C  The maximum difference density is > 0.1*ZMAX*0.75
            _refine_diff_density_max given =      3.130
            Test value =      2.625
DIFMX02_ALERT_1_C  The maximum difference density is > 0.1*ZMAX*0.75
            The relevant atom site should be identified.
RFACG01_ALERT_3_C  The value of the R factor is > 0.10
            R factor given   0.119
RFACR01_ALERT_3_C  The value of the weighted R factor is > 0.25
            Weighted R factor given   0.264
PLAT082_ALERT_2_C High R1 Value ................................      0.12 Report
PLAT084_ALERT_3_C High wR2 Value (i.e. > 0.25) ..................      0.26 Report
PLAT097_ALERT_2_C Large Reported Max.  (Positive) Residual Density      3.13 eA-3
PLAT098_ALERT_2_C Large Reported Min.  (Negative) Residual Density     -3.17 eA-3
PLAT341_ALERT_3_C Low Bond Precision on  C-C Bonds ..............     0.0104 Ang.
```

Residual electron density is located close to the bromide anions

Alert level G

```
PLAT002_ALERT_2_G Number of Distance or Angle Restraints on AtSite         78 Note
PLAT003_ALERT_2_G Number of Uiso or Uij Restrained non-H Atoms ...         78 Report
PLAT005_ALERT_5_G No _iucr_refine_instructions_details  in the CIF     Please Do !
PLAT007_ALERT_5_G Number of Unrefined Donor-H Atoms .............          12 Report
PLAT042_ALERT_1_G Calc. and Reported MoietyFormula Strings  Differ     Please Check
PLAT045_ALERT_1_G Calculated and Reported Z Differ by ...........        3.00 Ratio
PLAT063_ALERT_4_G Crystal Size Likely too Large for Beam Size ....       0.66 mm
PLAT112_ALERT_2_G ADDSYM Detects Additional (Pseudo) Symm. Elem...      sub Check
PLAT152_ALERT_1_G The Supplied and Calc. Volume s.u. Differ by ...         8 Units
PLAT606_ALERT_4_G VERY LARGE Solvent Accessible VOID(S) in Structure       ! Info
PLAT720_ALERT_4_G Number of Unusual/Non-Standard Labels .........         18 Note
PLAT808_ALERT_5_G No Parseable SHELXL Style Weighting Scheme Found     Please Check
PLAT860_ALERT_3_G Number of Least-Squares Restraints ............       1969 Note
PLAT869_ALERT_4_G ALERTS Related to the use of SQUEEZE Suppressed          ! Info
```

No additional symmetry could be found using PLATON ADDSYM or SUPERFLIP

```
  0 ALERT level A = Most likely a serious problem - resolve or explain
  1 ALERT level B = A potentially serious problem, consider carefully
 11 ALERT level C = Check. Ensure it is not caused by an omission or oversight
 14 ALERT level G = General information/check it is not something unexpected

  5 ALERT type 1 CIF construction/syntax error, inconsistent or missing data
  9 ALERT type 2 Indicator that the structure model may be wrong or deficient
  5 ALERT type 3 Indicator that the structure quality may be low
  4 ALERT type 4 Improvement, methodology, query or suggestion
  3 ALERT type 5 Informative message, check
```

Nanotubes prepared from **1**, where TBA cations have been modeled (see main Supporting Information document for more information)

Datablock: 1_partialsqueeze

```
Bond precision:   C-C = 0.0173 A          Wavelength=0.71073
```

Cell:	a=40.346(14)	b=40.346(14)	c=17.984(6)
	alpha=90	beta=90	gamma=120
Temperature:	90 K		

	Calculated	Reported
Volume	25352(23)	25352(15)
Space group	P 3 c 1	P 3 c 1
Hall group	P 3 -2"c	P 3 -2"c
Moiety formula	C22 H18 O4, 2(C16 H36 N), 2(Br)	3(C22 H18 O4), 6(C16 H36 N), 6(Br)
Sum formula	C54 H90 Br2 N2 O4	C162 H270 Br6 N6 O12
Mr	991.08	2973.36
Dx,g cm-3	1.168	1.168
Z	18	6
Mu (mm-1)	1.480	1.480
F000	9540.0	9540.0
F000'	9532.84	
h,k,lmax	50,50,22	43,50,22
Nref	34441[17233]	34142
Tmin,Tmax	0.593,0.661	0.623,1.000
Tmin'	0.373	

Correction method= # Reported T Limits: Tmin=0.623 Tmax=1.000
AbsCorr = MULTI-SCAN

Data completeness= 1.98/0.99   Theta(max)= 26.331

R(reflections)= 0.1799(29002)   wR2(reflections)= 0.3446(30931)

S = 0.987   Npar= 746

Due to the poor quality of the data, it was not possible to achieve a chemically-sensible anisotropic refinement of the data including modeling the TBA cations

---

The following ALERTS were generated. Each ALERT has the format
test-name_ALERT_alert-type_alert-level.
Click on the hyperlinks for more details of the test.

Alert level A

PLAT201_ALERT_2_A Isotropic non-H Atoms in Main Residue(s) .... 180 Report

---

Alert level B

```
DIFMN02_ALERT_2_B  The minimum difference density is < -0.1*ZMAX*1.00
            _refine_diff_density_min given =     -4.150
            Test value =     -3.500
DIFMX01_ALERT_2_B  The maximum difference density is > 0.1*ZMAX*1.00
            _refine_diff_density_max given =      6.630
            Test value =      3.500
RFACG01_ALERT_3_B  The value of the R factor is > 0.15
            R factor given    0.180
PLAT082_ALERT_2_B High R1 Value .................................      0.18 Report
PLAT097_ALERT_2_B Large Reported Max.  (Positive) Residual Density     6.63 eA-3
PLAT098_ALERT_2_B Large Reported Min.  (Negative) Residual Density    -4.15 eA-3
```

Residual electron density is located close to the bromide anions

```
PLAT341_ALERT_3_B Low Bond Precision on  C-C Bonds ...............     0.0173 Ang.
```

Alert level C

```
DIFMN03_ALERT_1_C  The minimum difference density is < -0.1*ZMAX*0.75
            The relevant atom site should be identified.
DIFMX02_ALERT_1_C  The maximum difference density is > 0.1*ZMAX*0.75
            The relevant atom site should be identified.
RFACR01_ALERT_3_C  The value of the weighted R factor is > 0.25
            Weighted R factor given   0.345
PLAT084_ALERT_3_C High wR2 Value (i.e. > 0.25) ..................       0.34 Report
PLAT202_ALERT_3_C Isotropic non-H Atoms in Anion/Solvent .........          6
PLAT758_ALERT_4_C D-H..A  Calc      130.00, Rep    130.3(4) ......  Senseless su
              C601 -H6012-O207    1.555    1.555    1.555      #       858
PLAT758_ALERT_4_C D-H..A  Calc      135.00, Rep    135.1(4) ......  Senseless su
              C401 -H4011-O1      1.555    1.555    1.555      #       858
PLAT758_ALERT_4_C D-H..A  Calc      146.00, Rep    145.7(4) ......  Senseless su
              C312 -H3122-O2      1.555    1.555    1.555      #       858
```

Alert level G

```
PLAT002_ALERT_2_G Number of Distance or Angle Restraints on AtSite        180 Note
PLAT003_ALERT_2_G Number of Uiso or Uij Restrained non-H Atoms ...        180 Report
PLAT005_ALERT_5_G No _iucr_refine_instructions_details  in the CIF     Please Do !
PLAT007_ALERT_5_G Number of Unrefined Donor-H Atoms ..............         12 Report
PLAT042_ALERT_1_G Calc. and Reported MoietyFormula Strings  Differ     Please Check
PLAT045_ALERT_1_G Calculated and Reported Z Differ by ............       3.00 Ratio
PLAT063_ALERT_4_G Crystal Size Likely too Large for Beam Size ....       0.66 mm
PLAT152_ALERT_1_G The Supplied and Calc. Volume s.u. Differ by ...          8 Units
PLAT606_ALERT_4_G VERY LARGE Solvent Accessible VOID(S) in Structure        ! Info
PLAT720_ALERT_4_G Number of Unusual/Non-Standard Labels ..........        252 Note
PLAT808_ALERT_5_G No Parseable SHELXL Style Weighting Scheme Found     Please Check
PLAT860_ALERT_3_G Number of Least-Squares Restraints .............        709 Note
PLAT869_ALERT_4_G ALERTS Related to the use of SQUEEZE Suppressed           ! Info
```

```
   1 ALERT level A = Most likely a serious problem - resolve or explain
   7 ALERT level B = A potentially serious problem, consider carefully
   8 ALERT level C = Check. Ensure it is not caused by an omission or oversight
  13 ALERT level G = General information/check it is not something unexpected

   5 ALERT type 1 CIF construction/syntax error, inconsistent or missing data
   8 ALERT type 2 Indicator that the structure model may be wrong or deficient
   6 ALERT type 3 Indicator that the structure quality may be low
   7 ALERT type 4 Improvement, methodology, query or suggestion
   3 ALERT type 5 Informative message, check
```

Datablock: oxidized_nanotubes_prepared_from_2

Nanotubes prepared from **2**, where oxidation of **2** occurred during crystal growth

```
Bond precision:   C-C = 0.0054 A          Wavelength=0.71073

Cell:             a=39.973(4)      b=39.973(4)      c=18.0631(18)
                  alpha=90         beta=90          gamma=120
Temperature:      90 K
```

	Calculated	Reported
Volume	24995(6)	24995(4)
Space group	R 3 c	R 3 c
Hall group	R 3 -2"c	R 3 -2"c
Moiety formula	C22 H16 O6, 2(C16 H36 N), 2(Br)	C22 H16 O6, 2(C16 H36 N), 2(Br)
Sum formula	C54 H88 Br2 N2 O6	C54 H88 Br2 N2 O6
Mr	1021.06	1021.11
Dx,g cm-3	1.221	1.221
Z	18	18
Mu (mm-1)	1.506	1.506
F000	9792.0	9792.0
F000'	9785.19	
h,k,lmax	51,51,23	44,51,23
Nref	12771[6398]	12725
Tmin,Tmax	0.655,0.697	0.705,1.000
Tmin'	0.325	

Correction method= # Reported T Limits: Tmin=0.705 Tmax=1.000
AbsCorr = MULTI-SCAN

Data completeness= 1.99/1.00 Theta(max)= 27.510

R(reflections)= 0.0506(11974) wR2(reflections)= 0.1030(12647)

S = 0.914 Npar= 731

The following ALERTS were generated. Each ALERT has the format
test-name_ALERT_alert-type_alert-level.
Click on the hyperlinks for more details of the test.

Alert level C

```
PLAT090_ALERT_3_C Poor Data / Parameter Ratio (Zmax > 18) ........       8.75 Note
PLAT213_ALERT_2_C Atom C134             has ADP max/min Ratio .....       3.3 oblate
PLAT213_ALERT_2_C Atom C233             has ADP max/min Ratio .....       3.8 oblate
PLAT213_ALERT_2_C Atom C234             has ADP max/min Ratio .....       3.2 oblate
PLAT222_ALERT_3_C Large Non-Solvent  H     Uiso(max)/Uiso(min) ...       4.3 Ratio
PLAT250_ALERT_2_C Large U3/U1 Ratio for Average U(i,j) Tensor ....       2.1 Note
PLAT250_ALERT_2_C Large U3/U1 Ratio for Average U(i,j) Tensor ....       2.1 Note
PLAT250_ALERT_2_C Large U3/U1 Ratio for Average U(i,j) Tensor ....       2.3 Note
PLAT413_ALERT_2_C Short Inter XH3 .. XHn     H742   ..  H2331    ..       2.05 Ang.
```

Alert level G

```
PLAT003_ALERT_2_G Number of Uiso or Uij Restrained non-H Atoms ...         51 Report
PLAT005_ALERT_5_G No _iucr_refine_instructions_details  in the CIF   Please Do !
PLAT007_ALERT_5_G Number of Unrefined Donor-H Atoms .............          4 Report
PLAT063_ALERT_4_G Crystal Size Likely too Large for Beam Size ....       0.74 mm
PLAT152_ALERT_1_G The Supplied and Calc. Volume s.u. Differ by ...          2 Units
PLAT300_ALERT_4_G Atom Site Occupancy of >N100   is Constrained at      0.650 Check
PLAT300_ALERT_4_G Atom Site Occupancy of >C101   is Constrained at      0.650 Check
PLAT300_ALERT_4_G Atom Site Occupancy of >C102   is Constrained at      0.650 Check
```

Major position of disordered TBA cation

```
PLAT300_ALERT_4_G Atom Site Occupancy of >C103   is Constrained at      0.650 Check
PLAT300_ALERT_4_G Atom Site Occupancy of >C104   is Constrained at      0.650 Check
PLAT300_ALERT_4_G Atom Site Occupancy of >C111   is Constrained at      0.650 Check
PLAT300_ALERT_4_G Atom Site Occupancy of >C112   is Constrained at      0.650 Check
PLAT300_ALERT_4_G Atom Site Occupancy of >C113   is Constrained at      0.650 Check
PLAT300_ALERT_4_G Atom Site Occupancy of >C114   is Constrained at      0.650 Check
PLAT300_ALERT_4_G Atom Site Occupancy of >C121   is Constrained at      0.650 Check
PLAT300_ALERT_4_G Atom Site Occupancy of >C122   is Constrained at      0.650 Check
PLAT300_ALERT_4_G Atom Site Occupancy of >C123   is Constrained at      0.650 Check
PLAT300_ALERT_4_G Atom Site Occupancy of >C124   is Constrained at      0.650 Check
PLAT300_ALERT_4_G Atom Site Occupancy of >C131   is Constrained at      0.650 Check
PLAT300_ALERT_4_G Atom Site Occupancy of >C132   is Constrained at      0.650 Check
PLAT300_ALERT_4_G Atom Site Occupancy of >C133   is Constrained at      0.650 Check
PLAT300_ALERT_4_G Atom Site Occupancy of >C134   is Constrained at      0.650 Check
PLAT300_ALERT_4_G Atom Site Occupancy of >H1012  is Constrained at      0.650 Check
PLAT300_ALERT_4_G Atom Site Occupancy of >H1011  is Constrained at      0.650 Check
PLAT300_ALERT_4_G Atom Site Occupancy of >H1111  is Constrained at      0.650 Check
PLAT300_ALERT_4_G Atom Site Occupancy of >H1112  is Constrained at      0.650 Check
PLAT300_ALERT_4_G Atom Site Occupancy of >H1121  is Constrained at      0.650 Check
PLAT300_ALERT_4_G Atom Site Occupancy of >H1122  is Constrained at      0.650 Check
PLAT300_ALERT_4_G Atom Site Occupancy of >H1131  is Constrained at      0.650 Check
PLAT300_ALERT_4_G Atom Site Occupancy of >H1132  is Constrained at      0.650 Check
PLAT300_ALERT_4_G Atom Site Occupancy of >H1141  is Constrained at      0.650 Check
PLAT300_ALERT_4_G Atom Site Occupancy of >H1143  is Constrained at      0.650 Check
PLAT300_ALERT_4_G Atom Site Occupancy of >H1142  is Constrained at      0.650 Check
PLAT300_ALERT_4_G Atom Site Occupancy of >H1211  is Constrained at      0.650 Check
PLAT300_ALERT_4_G Atom Site Occupancy of >H1212  is Constrained at      0.650 Check
PLAT300_ALERT_4_G Atom Site Occupancy of >H1221  is Constrained at      0.650 Check
PLAT300_ALERT_4_G Atom Site Occupancy of >H1222  is Constrained at      0.650 Check
PLAT300_ALERT_4_G Atom Site Occupancy of >H1231  is Constrained at      0.650 Check
PLAT300_ALERT_4_G Atom Site Occupancy of >H1232  is Constrained at      0.650 Check
PLAT300_ALERT_4_G Atom Site Occupancy of >H1241  is Constrained at      0.650 Check
PLAT300_ALERT_4_G Atom Site Occupancy of >H1242  is Constrained at      0.650 Check
PLAT300_ALERT_4_G Atom Site Occupancy of >H1243  is Constrained at      0.650 Check
PLAT300_ALERT_4_G Atom Site Occupancy of >H1311  is Constrained at      0.650 Check
PLAT300_ALERT_4_G Atom Site Occupancy of >H1312  is Constrained at      0.650 Check
PLAT300_ALERT_4_G Atom Site Occupancy of >H1321  is Constrained at      0.650 Check
PLAT300_ALERT_4_G Atom Site Occupancy of >H1322  is Constrained at      0.650 Check
PLAT300_ALERT_4_G Atom Site Occupancy of >H1332  is Constrained at      0.650 Check
PLAT300_ALERT_4_G Atom Site Occupancy of >H1331  is Constrained at      0.650 Check
PLAT300_ALERT_4_G Atom Site Occupancy of >H1341  is Constrained at      0.650 Check
PLAT300_ALERT_4_G Atom Site Occupancy of >H1343  is Constrained at      0.650 Check
PLAT300_ALERT_4_G Atom Site Occupancy of >H1342  is Constrained at      0.650 Check
PLAT300_ALERT_4_G Atom Site Occupancy of >H1021  is Constrained at      0.650 Check
PLAT300_ALERT_4_G Atom Site Occupancy of >H1022  is Constrained at      0.650 Check
PLAT300_ALERT_4_G Atom Site Occupancy of >H1031  is Constrained at      0.650 Check
PLAT300_ALERT_4_G Atom Site Occupancy of >H1032  is Constrained at      0.650 Check
PLAT300_ALERT_4_G Atom Site Occupancy of >H1042  is Constrained at      0.650 Check
PLAT300_ALERT_4_G Atom Site Occupancy of >H1041  is Constrained at      0.650 Check
PLAT300_ALERT_4_G Atom Site Occupancy of >H1043  is Constrained at      0.650 Check
PLAT300_ALERT_4_G Atom Site Occupancy of <N200   is Constrained at      0.350 Check
PLAT300_ALERT_4_G Atom Site Occupancy of <C201   is Constrained at      0.350 Check
PLAT300_ALERT_4_G Atom Site Occupancy of <C202   is Constrained at      0.350 Check
PLAT300_ALERT_4_G Atom Site Occupancy of <C203   is Constrained at      0.350 Check
PLAT300_ALERT_4_G Atom Site Occupancy of <C204   is Constrained at      0.350 Check
PLAT300_ALERT_4_G Atom Site Occupancy of <C211   is Constrained at      0.350 Check
PLAT300_ALERT_4_G Atom Site Occupancy of <C212   is Constrained at      0.350 Check
PLAT300_ALERT_4_G Atom Site Occupancy of <C213   is Constrained at      0.350 Check
PLAT300_ALERT_4_G Atom Site Occupancy of <C214   is Constrained at      0.350 Check
PLAT300_ALERT_4_G Atom Site Occupancy of <C221   is Constrained at      0.350 Check
PLAT300_ALERT_4_G Atom Site Occupancy of <C222   is Constrained at      0.350 Check
PLAT300_ALERT_4_G Atom Site Occupancy of <C223   is Constrained at      0.350 Check
```

Major position of disordered TBA cation

Minor position of disordered TBA cation

```
PLAT300_ALERT_4_G Atom Site Occupancy of <C224   is Constrained at      0.350 Check
PLAT300_ALERT_4_G Atom Site Occupancy of <C231   is Constrained at      0.350 Check
PLAT300_ALERT_4_G Atom Site Occupancy of <C232   is Constrained at      0.350 Check
PLAT300_ALERT_4_G Atom Site Occupancy of <C233   is Constrained at      0.350 Check
PLAT300_ALERT_4_G Atom Site Occupancy of <C234   is Constrained at      0.350 Check
PLAT300_ALERT_4_G Atom Site Occupancy of <H2021  is Constrained at      0.350 Check
PLAT300_ALERT_4_G Atom Site Occupancy of <H2022  is Constrained at      0.350 Check
PLAT300_ALERT_4_G Atom Site Occupancy of <H2031  is Constrained at      0.350 Check
PLAT300_ALERT_4_G Atom Site Occupancy of <H2032  is Constrained at      0.350 Check
PLAT300_ALERT_4_G Atom Site Occupancy of <H2041  is Constrained at      0.350 Check
PLAT300_ALERT_4_G Atom Site Occupancy of <H2043  is Constrained at      0.350 Check
PLAT300_ALERT_4_G Atom Site Occupancy of <H2042  is Constrained at      0.350 Check
PLAT300_ALERT_4_G Atom Site Occupancy of <H2012  is Constrained at      0.350 Check
PLAT300_ALERT_4_G Atom Site Occupancy of <H2011  is Constrained at      0.350 Check
PLAT300_ALERT_4_G Atom Site Occupancy of <H2112  is Constrained at      0.350 Check
PLAT300_ALERT_4_G Atom Site Occupancy of <H2111  is Constrained at      0.350 Check
PLAT300_ALERT_4_G Atom Site Occupancy of <H2121  is Constrained at      0.350 Check
PLAT300_ALERT_4_G Atom Site Occupancy of <H2122  is Constrained at      0.350 Check
PLAT300_ALERT_4_G Atom Site Occupancy of <H2131  is Constrained at      0.350 Check
PLAT300_ALERT_4_G Atom Site Occupancy of <H2132  is Constrained at      0.350 Check
PLAT300_ALERT_4_G Atom Site Occupancy of <H2141  is Constrained at      0.350 Check
PLAT300_ALERT_4_G Atom Site Occupancy of <H2143  is Constrained at      0.350 Check
PLAT300_ALERT_4_G Atom Site Occupancy of <H2142  is Constrained at      0.350 Check
PLAT300_ALERT_4_G Atom Site Occupancy of <H2211  is Constrained at      0.350 Check
PLAT300_ALERT_4_G Atom Site Occupancy of <H2212  is Constrained at      0.350 Check
PLAT300_ALERT_4_G Atom Site Occupancy of <H2221  is Constrained at      0.350 Check
PLAT300_ALERT_4_G Atom Site Occupancy of <H2222  is Constrained at      0.350 Check
PLAT300_ALERT_4_G Atom Site Occupancy of <H2232  is Constrained at      0.350 Check
PLAT300_ALERT_4_G Atom Site Occupancy of <H2231  is Constrained at      0.350 Check
PLAT300_ALERT_4_G Atom Site Occupancy of <H2242  is Constrained at      0.350 Check
PLAT300_ALERT_4_G Atom Site Occupancy of <H2241  is Constrained at      0.350 Check
PLAT300_ALERT_4_G Atom Site Occupancy of <H2243  is Constrained at      0.350 Check
PLAT300_ALERT_4_G Atom Site Occupancy of <H2312  is Constrained at      0.350 Check
PLAT300_ALERT_4_G Atom Site Occupancy of <H2311  is Constrained at      0.350 Check
PLAT300_ALERT_4_G Atom Site Occupancy of <H2322  is Constrained at      0.350 Check
PLAT300_ALERT_4_G Atom Site Occupancy of <H2321  is Constrained at      0.350 Check
PLAT300_ALERT_4_G Atom Site Occupancy of <H2332  is Constrained at      0.350 Check
PLAT300_ALERT_4_G Atom Site Occupancy of <H2331  is Constrained at      0.350 Check
PLAT300_ALERT_4_G Atom Site Occupancy of <H2341  is Constrained at      0.350 Check
PLAT300_ALERT_4_G Atom Site Occupancy of <H2343  is Constrained at      0.350 Check
PLAT300_ALERT_4_G Atom Site Occupancy of <H2342  is Constrained at      0.350 Check
PLAT301_ALERT_3_G Main Residue  Disorder ........... Percentage =         27 Note
PLAT304_ALERT_4_G Non-Integer Number of Atoms (   34.45) in Resd. #         3 Check
PLAT304_ALERT_4_G Non-Integer Number of Atoms (   18.55) in Resd. #         4 Check
PLAT333_ALERT_2_G Check Large Av C6-Ring C-C Dist. C13     -C18          1.44 Ang.
PLAT335_ALERT_2_G Check Large C6 Ring     C-C Range C13     -C18          0.17 Ang.
PLAT605_ALERT_4_G Structure Contains Solvent Accessible VOIDS of .        385 A**3
PLAT720_ALERT_4_G Number of Unusual/Non-Standard Labels ..........         72 Note
PLAT808_ALERT_5_G No Parseable SHELXL Style Weighting Scheme Found     Please Check
PLAT811_ALERT_5_G No ADDSYM Analysis: Too Many Excluded Atoms ....          ! Info
PLAT860_ALERT_3_G Number of Least-Squares Restraints .............        391 Note
PLAT869_ALERT_4_G ALERTS Related to the use of SQUEEZE Suppressed           ! Info
```

```
  0 ALERT level A = Most likely a serious problem - resolve or explain
  0 ALERT level B = A potentially serious problem, consider carefully
  9 ALERT level C = Check. Ensure it is not caused by an omission or oversight
122 ALERT level G = General information/check it is not something unexpected

  1 ALERT type 1 CIF construction/syntax error, inconsistent or missing data
```

Minor position of disordered TBA cation

Due to 0.65/0.35 occupancies of TBA cation disorder (these values sum to 53 atoms, for $C_{16}H_{36}N$)

These alerts are caused by the quinone ring (which has a large average C-C bond length, and a wide range of bond lengths due to both single and double bonds)

```
 10 ALERT type 2 Indicator that the structure model may be wrong or deficient
  4 ALERT type 3 Indicator that the structure quality may be low
112 ALERT type 4 Improvement, methodology, query or suggestion
  4 ALERT type 5 Informative message, check
```

Datablock: oxidized_nanotubes_prepared_from_3

Nanotubes prepared from **3**

```
Bond precision:   C-C = 0.0108 A          Wavelength=0.71073

Cell:             a=39.896(4)      b=39.896(4)      c=18.0796(17)
                  alpha=90         beta=90          gamma=120
Temperature:      90 K
```

	Calculated	Reported
Volume	24922(6)	24921(4)
Space group	R 3 c	R 3 c
Hall group	R 3 -2"c	R 3 -2"c
Moiety formula	C22 H16 O6, 2(C16 H36 N), 2(Br)	C22 H16 O6, 2(Br), 2(C16 H36 N)
Sum formula	C54 H88 Br2 N2 O6	C54 H88 Br2 N2 O6
Mr	1021.06	1021.11
Dx,g cm-3	1.225	1.225
Z	18	18
Mu (mm-1)	1.510	1.510
F000	9792.0	9791.9
F000’	9785.19	
h,k,lmax	49,49,22	42,49,22
Nref	11356[5690]	10976
Tmin,Tmax	0.865,0.956	0.889,1.000
Tmin’	0.547	

```
Correction method= # Reported T Limits: Tmin=0.889 Tmax=1.000
AbsCorr = MULTI-SCAN

Data completeness= 1.93/0.97       Theta(max)= 26.388

R(reflections)= 0.0573( 6687)      wR2(reflections)= 0.1415( 8235)

S = 0.951                   Npar= 578
```

The following ALERTS were generated. Each ALERT has the format
test-name_ALERT_alert-type_alert-level.
Click on the hyperlinks for more details of the test.

Alert level B

```
PLAT250_ALERT_2_B Large U3/U1 Ratio for Average U(i,j) Tensor ....        4.9 Note
```

Alert level C

```
PLAT090_ALERT_3_C Poor Data / Parameter Ratio (Zmax > 18) ........       9.84 Note
PLAT213_ALERT_2_C Atom N150            has ADP max/min Ratio .....        3.4 prolat
PLAT213_ALERT_2_C Atom C161            has ADP max/min Ratio .....        3.5 prolat
PLAT213_ALERT_2_C Atom C181            has ADP max/min Ratio .....        3.1 prolat
PLAT220_ALERT_2_C Large Non-Solvent  C     Ueq(max)/Ueq(min) Range        3.4 Ratio
PLAT222_ALERT_3_C Large Non-Solvent  H     Uiso(max)/Uiso(min) ...        4.1 Ratio
PLAT241_ALERT_2_C High      Ueq as Compared to Neighbors for .....       C133 Check
PLAT241_ALERT_2_C High      Ueq as Compared to Neighbors for .....       C153 Check
PLAT241_ALERT_2_C High      Ueq as Compared to Neighbors for .....       C172 Check
PLAT242_ALERT_2_C Low       Ueq as Compared to Neighbors for .....       C132 Check
PLAT242_ALERT_2_C Low       Ueq as Compared to Neighbors for .....       C152 Check
PLAT242_ALERT_2_C Low       Ueq as Compared to Neighbors for .....       C173 Check
PLAT341_ALERT_3_C Low Bond Precision on  C-C Bonds ..............     0.0108 Ang.
PLAT758_ALERT_4_C D-H..A  Calc      133.00, Rep    133.4(2) ......  Senseless su
              C101 -H1012-O8      1.555    1.555    1.555      #        286
```

Alert level G

```
PLAT002_ALERT_2_G Number of Distance or Angle Restraints on AtSite         34 Note
PLAT003_ALERT_2_G Number of Uiso or Uij Restrained non-H Atoms ...         34 Report
PLAT005_ALERT_5_G No _iucr_refine_instructions_details  in the CIF     Please Do !
PLAT007_ALERT_5_G Number of Unrefined Donor-H Atoms ..............          4 Report
PLAT042_ALERT_1_G Calc. and Reported MoietyFormula Strings  Differ     Please Check
PLAT152_ALERT_1_G The Supplied and Calc. Volume s.u. Differ by ...          2 Units
PLAT333_ALERT_2_G Check Large Av C6-Ring C-C Dist. C13    -C18           1.43 Ang.
PLAT335_ALERT_2_G Check Large C6 Ring    C-C Range C13    -C18           0.18 Ang.
PLAT605_ALERT_4_G Structure Contains Solvent Accessible VOIDS of .        429 A**3
PLAT720_ALERT_4_G Number of Unusual/Non-Standard Labels ..........         72 Note
PLAT808_ALERT_5_G No Parseable SHELXL Style Weighting Scheme Found     Please Check
PLAT860_ALERT_3_G Number of Least-Squares Restraints .............        293 Note
PLAT869_ALERT_4_G ALERTS Related to the use of SQUEEZE Suppressed           ! Info
```

```
   0 ALERT level A = Most likely a serious problem - resolve or explain
   1 ALERT level B = A potentially serious problem, consider carefully
  14 ALERT level C = Check. Ensure it is not caused by an omission or oversight
  13 ALERT level G = General information/check it is not something unexpected

   2 ALERT type 1 CIF construction/syntax error, inconsistent or missing data
  15 ALERT type 2 Indicator that the structure model may be wrong or deficient
   4 ALERT type 3 Indicator that the structure quality may be low
   4 ALERT type 4 Improvement, methodology, query or suggestion
   3 ALERT type 5 Informative message, check
```

These alerts are caused by the quinone ring (which has a large average C-C bond length, and a wide range of bond lengths due to both single and double bonds)

It is advisable to attempt to resolve as many as possible of the alerts in all categories. Often the minor alerts point to easily fixed oversights, errors and omissions in your CIF or refinement strategy, so attention to these fine details can be worthwhile. In order to resolve some of the more serious problems it may be necessary to carry out additional measurements or structure refinements. However, the purpose of your study may justify the reported deviations and the more serious of these should normally be commented upon in the discussion or experimental section of a paper or in the "special_details" fields of the CIF. checkCIF was carefully designed to identify outliers and unusual parameters, but every test has its limitations and alerts that are not important in a particular case may appear. Conversely, the absence of alerts does not guarantee there are no aspects of the results needing attention. It is up to the individual to critically assess their own results and, if necessary, seek expert advice.

Publication of your CIF in IUCr journals

A basic structural check has been run on your CIF. These basic checks will be run on all CIFs submitted for publication in IUCr journals (*Acta Crystallographica*, *Journal of Applied Crystallography*, *Journal of Synchrotron Radiation*); however, if you intend to submit to *Acta Crystallographica Section C* or *E*, you should make sure that full publication checks are run on the final version of your CIF prior to submission.

Publication of your CIF in other journals

Please refer to the *Notes for Authors* of the relevant journal for any special instructions relating to CIF submission.

PLATON version of 21/06/2015; check.def file version of 21/06/2015

Datablock 1_fullsqueeze - ellipsoid plot

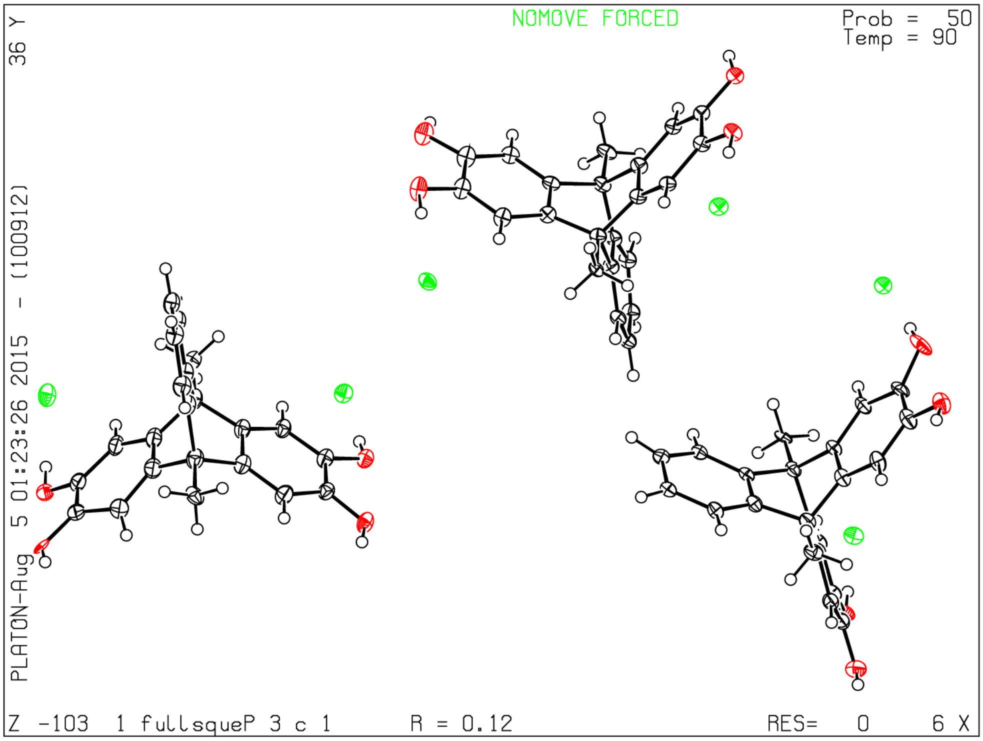

Datablock 1_partialsqueeze - ellipsoid plot

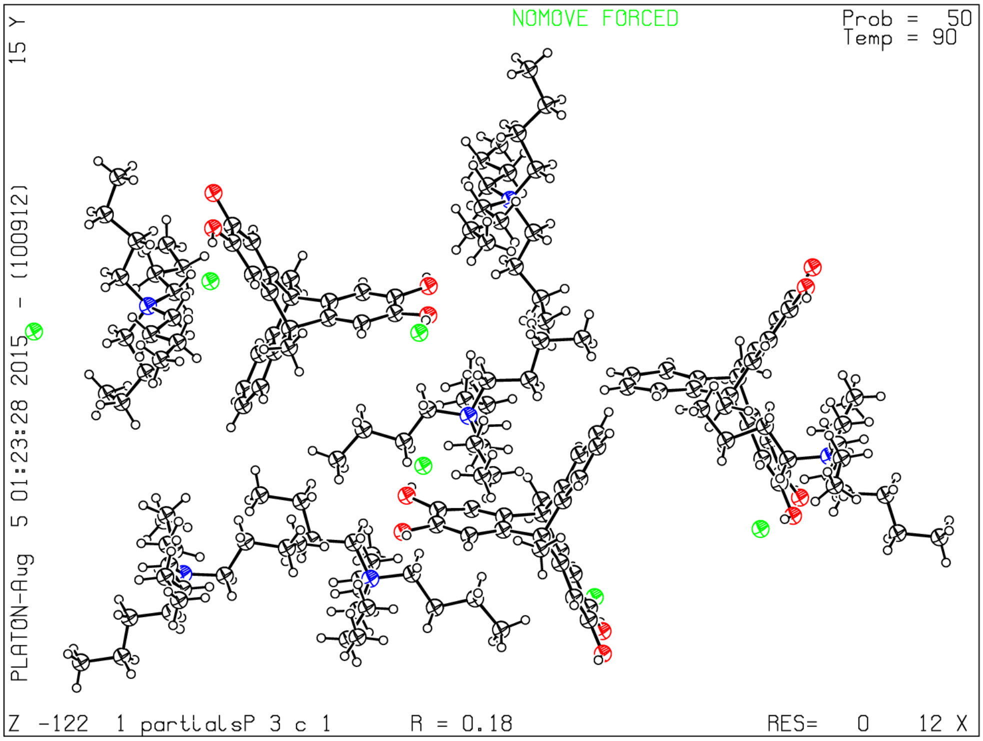

Datablock oxidized_nanotubes_prepared_from_2 - ellipsoid plot

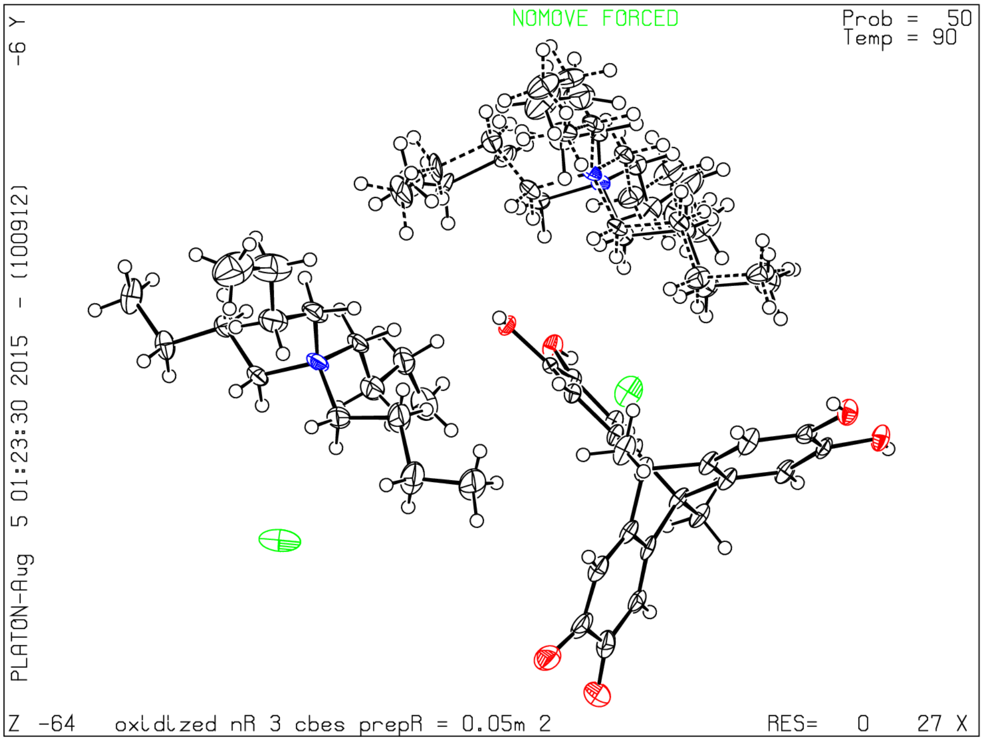

Datablock oxidized_nanotubes_prepared_from_3 - ellipsoid plot

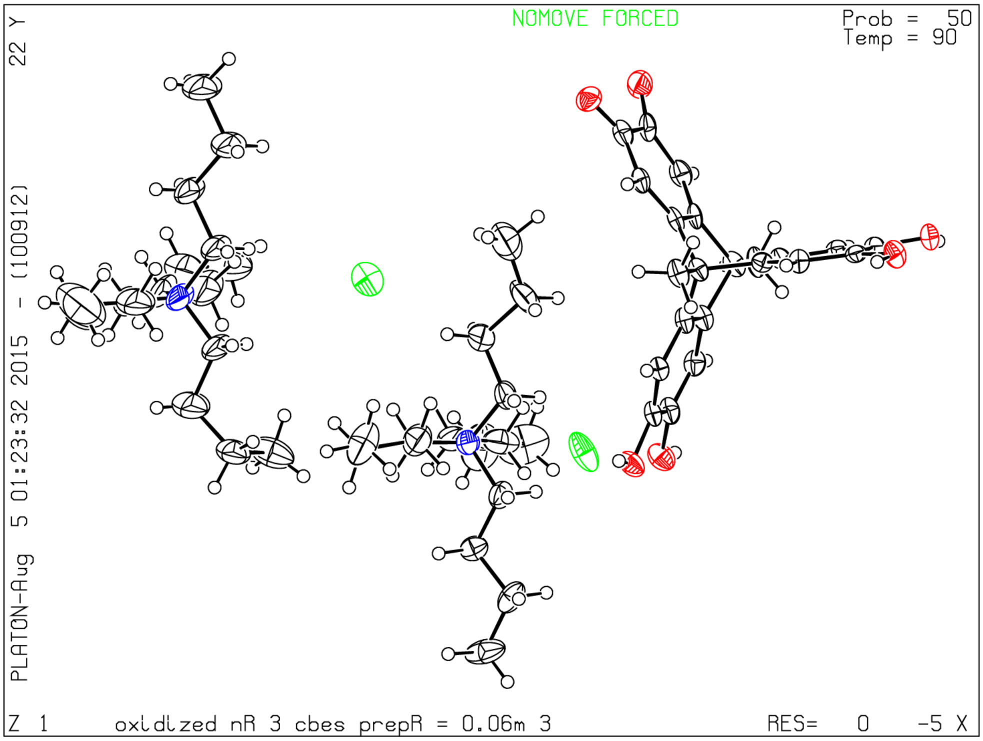

Supplement: Supplementary file 1 [file SC-006-C5SC02577J-s001.zip › Annotated checkCIF.pdf]
